# Creating correct blur and its effect on accommodation

**DOI:** 10.1167/18.9.1

**Published:** 2018-09-04

**Authors:** Steven A. Cholewiak, Gordon D. Love, Martin S. Banks

**Affiliations:** steven.cholewiak@berkeley.edu http://steven.cholewiak.com; g.d.love@durham.ac.uk https://www.dur.ac.uk/computer.science/staff/profile/?id=246; martybanks@berkeley.edu http://bankslab.berkeley.edu; Optometry & Vision Science, University of California, Berkeley, Berkeley, CA, USA; Computer Science & Physics, Durham University, Durham, UK; Optometry & Vision Science, University of California, Berkeley, Berkeley, CA, USA

**Keywords:** chromatic aberration, accommodation, vergence-accommodation conflict, head-mounted displays, lca

## Abstract

Blur occurs naturally when the eye is focused at one distance and an object is presented at another distance. Computer-graphics engineers and vision scientists often wish to create display images that reproduce such depth-dependent blur, but their methods are incorrect for that purpose. They take into account the scene geometry, pupil size, and focal distances, but do not properly take into account the optical aberrations of the human eye. We developed a method that, by incorporating the viewer's optics, yields displayed images that produce retinal images close to the ones that occur in natural viewing. We concentrated on the effects of defocus, chromatic aberration, astigmatism, and spherical aberration and evaluated their effectiveness by conducting experiments in which we attempted to drive the eye's focusing response (accommodation) through the rendering of these aberrations. We found that accommodation is not driven at all by conventional rendering methods, but that it is driven surprisingly quickly and accurately by our method with defocus and chromatic aberration incorporated. We found some effect of astigmatism but none of spherical aberration. We discuss how the rendering approach can be used in vision science experiments and in the development of ophthalmic/optometric devices and augmented- and virtual-reality displays.

## Introduction

Rendering in computer graphics has focused on “photorealism”: simulating images from a camera with a pinhole or idealized lenses without aberrations. Even simulations of realistic optics have focused on ray-tracing camera lenses with well-corrected aberrations (Kolb, Mitchell, & Hanrahan, 1995; Ng & Hanrahan, 2006; Steinert, Dammertz, Hanika, & Lensch, 2011). It is important to note that such rendering techniques do not produce the retinal images that are produced by typical human eyes viewing natural scenes because they do not take into account the eye's imperfect optics. For example, consider an eye with longitudinal chromatic aberration (LCA) that is focused on a white point on a dark background. If the eye focuses medium wavelengths (e.g., green), short and long wavelengths will be focused respectively in front of and behind the retina, and will therefore appear blurred (Thibos, Ye, Zhang, & Bradley, 1992). If the white point is now moved closer to the eye (and the eye does not accommodate), short wavelengths will be focused closer to the retina and will appear sharper than before, and long wavelengths will be focused farther from behind the retina and will appear blurrier than before. This depth-dependent effect is not replicated in conventional photorealistic rendering.

A similar issue arises in most of the vision-science literature. Blur is rendered into visual stimuli to simulate different object depths, but depth-dependent effects—e.g., LCA, astigmatism, spherical aberration—are not incorporated. The blur kernel is usually an isotropic 2 D Gaussian with all wavelengths treated the same (Watson & Ahumada, 2011).

In a previous paper, we described how to implement rendering that incorporates natural aberrations (Cholewiak et al., 2017). This involves calculating what the retinal image should be and then computing the image to display that, when processed through the viewer's aberrated eye, creates the intended, natural retinal image. We described some preliminary experiments that showed that incorporation of one depth-dependent optical effect (chromatic aberration) enables one to drive accommodation. In the current paper, we describe a simpler rendering method and examine in more detail how the resulting displayed images can be used to drive accommodation.

## Accommodation

Accommodation is a control system that adjusts the power of the eye's crystalline lens to maintain a sharp retinal image. In control systems, an error signal with magnitude and sign information is an odd-error signal (Eykhoff, 1972). A signal with magnitude but no sign information is even-error. Blur due to defocus is an even-error signal: identical whether the object creating the out-of-focus image is farther or nearer than where the eye is accommodated. If only defocus blur were used to guide accommodation, the initial direction of corrective response could only be random. But after the initial response, the system could determine if blur has increased or decreased, and then adjust the lens to eventually attain a sharp retinal image. Thus, even-error signals would necessarily produce trial-and-error behavior as when focusing a microscope or binoculars (Stark & Takahashi, 1965).

The even-error problem in accommodation might be solved under everyday circumstances by using other depth information such as binocular vergence. When an object draws nearer, the eyes must converge to maintain a single image, and the vergence response can drive accommodation in the right direction (Cumming & Judge, 1986; Schor, 1992). Many other depth cues (size change, occlusion, motion parallax, texture gradient, etc.) could also provide useful information. This point was made by Stark and Takahashi (1965):

This lack of a simple optical odd-error signal may be of little functional significance in everyday vision. In such a situation a person usually accommodates in association with convergence, while perceiving a highly structured image with many reinforcing perceptual clues and accommodates in a direction which may also have been predicted by the recent past history of the spatial environment. (p. 146)

This argument is misleading. Vergence and the other cues are ancillary cues for accommodation: They are correlated with the changes in focal distance to which the system responds. But none of them can guide accommodation accurately because they cannot directly specify whether the retinal image is sharply focused or not. An example helps make this point. Imagine a control system for maintaining the temperature of a room. When the temperature is too low, the thermostat senses it, and the heater is activated until the temperature has reached the desired value. When temperature is too high, the air conditioner is turned on, and so forth. There are many ancillary cues that are predictive of the adjustment that will be needed. For example, the clock can inform the system that it is nighttime, so the heater should be turned on. But none of these ancillary cues would be used by themselves to control the room environment because they are too inaccurate as predictors. Rather the control variable should be the temperature measured by a thermometer. In general, control systems work best as negative-feedback systems in which the effector is constantly adjusted in the face of varying input to attain a target output value. Without such a negative-feedback loop, changes in the relationships between the input, effector, and output lead to erroneous adjustments. The accommodative system could never become properly calibrated to maintain image sharpness if it relied only on the above ancillary cues because none of them are directly informative of the lens power required to maximize sharpness. For this reason, the system should create negative feedback by monitoring retinal-image sharpness because this allows it to recalibrate as the eye grows, the pupil changes size, the lens becomes less elastic, etc. It is not surprising then to find that the eye does in fact accommodate accurately when ancillary depth cues are stripped away.

This was demonstrated elegantly by Smithline (1974). He presented a stimulus in best focus and then stepped it nearer or farther from the eye and measured the accommodative response. The stimulus was viewed monocularly, so binocular disparity could not be used to determine the direction of change. Head position was fixed, so motion parallax could not be used. The stimulus was presented in a Badal lens system, so there was no change in image size. The only informative signal was the blur of the retinal image. Strikingly, the first measurable response was always in the right direction: The eye knew the direction it needed to go to refocus the image. We will refer to this as the *Optical Blur* condition. Smithline then calculated the blur caused by the change in stimulus distance and inserted that blur into the stimulus by introducing a diffusion screen. In this case, of course, no accommodative response could restore the retinal image to its initial clarity. We will refer to this as the *Rendered Blur* condition. Interestingly, Smithline now observed no response, which means that the eye knew that nothing could be gained by changing focus when the blur was rendered rather than optically induced. These results show that with optically induced blur the retinal image contains odd-error information that is used to solve the problem of the direction and magnitude of response required to maximize retinal-image sharpness. The results have stood the test of time (Kruger, Mathews, Aggarwala, & Sanchez, 1993; Kruger, Mathews, Aggarwala, Yager, & Kruger, 1995; Stone, Mathews, & Kruger, 1993; Chen, Kruger, Hofer, Singer, & Williams, 2006).

Three image-based signals could potentially provide odd-error information: higher-order aberrations, micro-fluctuations, and longitudinal chromatic aberration. We describe these signals below, but first describe the properties of defocus.

### Defocus

Defocus is caused by the eye being focused at a different distance than an object. Presumably, the role of accommodation is to minimize defocus. The point-spread function (PSF) due to defocus alone (ignoring diffraction) is a cylinder whose diameter depends on the diameter of the viewer's pupil and where the eye is focused relative to the object causing defocus. The PSF diameter (for one wavelength) is given to close approximation by

**Figure 1:**
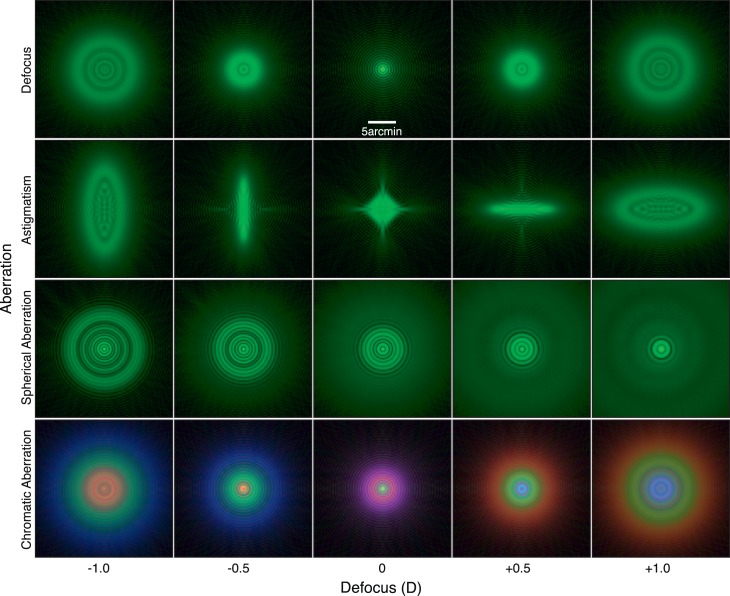
Retinal point-spread functions (PSFs) for defocus alone and defocus plus specific aberrations. Defocus from left to right is −1.0 to +1.0 D (hyperopic focus is negative). Pupil diameter is 4 mm; wavelength is 520 nm for the top three rows and 449, 520, and 617 nm for the bottom row. Diffraction is included. Top row: Defocus plus diffraction. Second row: Astigmatism with magnitude of 0.5 D and axis of 180°. Third row: Spherical aberration of 0.29μm. Bottom row: Longitudinal chromatic aberration. Intensities have been gamma expanded (γ = 5) to aid visibility.

\newcommand{\bialpha}{\boldsymbol{\alpha}}\newcommand{\bibeta}{\boldsymbol{\beta}}\newcommand{\bigamma}{\boldsymbol{\gamma}}\newcommand{\bidelta}{\boldsymbol{\delta}}\newcommand{\bivarepsilon}{\boldsymbol{\varepsilon}}\newcommand{\bizeta}{\boldsymbol{\zeta}}\newcommand{\bieta}{\boldsymbol{\eta}}\newcommand{\bitheta}{\boldsymbol{\theta}}\newcommand{\biiota}{\boldsymbol{\iota}}\newcommand{\bikappa}{\boldsymbol{\kappa}}\newcommand{\bilambda}{\boldsymbol{\lambda}}\newcommand{\bimu}{\boldsymbol{\mu}}\newcommand{\binu}{\boldsymbol{\nu}}\newcommand{\bixi}{\boldsymbol{\xi}}\newcommand{\biomicron}{\boldsymbol{\micron}}\newcommand{\bipi}{\boldsymbol{\pi}}\newcommand{\birho}{\boldsymbol{\rho}}\newcommand{\bisigma}{\boldsymbol{\sigma}}\newcommand{\bitau}{\boldsymbol{\tau}}\newcommand{\biupsilon}{\boldsymbol{\upsilon}}\newcommand{\biphi}{\boldsymbol{\phi}}\newcommand{\bichi}{\boldsymbol{\chi}}\newcommand{\bipsi}{\boldsymbol{\psi}}\newcommand{\biomega}{\boldsymbol{\omega}}\begin{equation}\tag{1}\beta = A\left| {{1 \over {z_0}} - {1 \over {z_1}}} \right| = A\left| {\Delta D} \right|\end{equation}

where *β* is in radians, *A* is pupil diameter in meters, *z*_0_ is distance to which the eye is focused, *z*_1_ is distance to the object creating the blurred image, and Δ*D* is the difference in those distances in diopters (Held, Cooper, O'Brien, & Banks, 2010). Importantly, the PSF due to defocus alone is identical whether the object is farther or nearer than the eye's current focus distance. This is illustrated in Figure 1, which shows the PSFs due to defocus plus diffraction for various accommodative errors. They are identical for positive and negative errors. Thus, appropriate rendering of defocus alone is the same for far and near parts of the scene.

### Higher-order aberrations and astigmatism

The eye's optical imperfections (apart from diffraction and scatter) can be described by deviations from a perfect sphere of the wavefront exiting the eye's focusing elements and converging on the retina. Those deviations are generally classified by Zernike polynomials. In most eyes, the Zernike terms of defocus and astigmatism constitute over 90% of the total deviation from an ideal optical system (Porter, Guirao, Cox, & Williams, 2001; Plainis & Pallikaris, 2008). The other terms are higher order aberrations (HOAs) and are quite small and possibly insignificant.

#### Astigmatism

Astigmatism occurs when rays propagating in perpendicular planes through the eye are focused at different distances. Measurable astigmatism occurs in the majority of adults (Satterfield, 1989). Its magnitude and axis vary across individuals. In most people the magnitude is not large enough to warrant optical correction (Katz, Tielsch, & Sommer, 1997). The PSF of a defocused astigmatic eye is elliptical with the major axis in one direction when the object is farther than current focus and with the major axis rotated by 90° when the object is nearer than current focus (Figure 1). Thus, astigmatism could provide odd-error information to help guide accommodation in the correct direction.

#### Spherical aberration

A perfect optical system focuses central and peripheral rays to a point in the image. When accommodated far, human eyes exhibit positive spherical aberration: Peripheral rays are focused in front of the retina when central rays are focused at the retina (Cheng et al., 2004; López-Gil et al., 2007). Spherical aberration thus creates different PSFs for objects farther as opposed to nearer than the eye's current focus (Figure 1; Wilson, Decker, & Roorda, 2002; Thibos, Bradley, Liu, & López-Gil, 2013). This signal could therefore provide odd-error information to guide accommodation in the right direction. As the eye accommodates nearer, spherical aberration shifts toward negative values (Cheng et al., 2004; Tarrant, Roorda, & Wildsoet, 2010).

Some research has investigated whether HOAs are used to drive accommodation. Most have compared accommodation with and without correction of various HOAs. The results are inconclusive. Fernández and Artal (2005) compared responses when HOAs were not corrected to responses when all HOAs (except spherical aberration) were corrected. Correction had essentially no effect. Gambra, Sawides, Dorronsoro, and Marcos (2009) also compared responses with and without HOA correction and found that some subjects exhibited slightly more accurate accommodation when HOAs were corrected. Chen et al. (2006) also compared responses with and without correction. One subject could not accommodate in any condition, four were able to accommodate but were unaffected by HOA correction, and one was able to accommodate but responses were less accurate and slower with HOAs corrected. Chin, Hampson, and Mallen (2009a) measured responses with various combinations of HOAs corrected or not. One of five subjects exhibited degradation of accommodation in one of four conditions in which HOAs were fully or partially corrected. Chin, Hampson, and Mallen (2009b) examined responses when the even-order Zernike terms (providing odd-error signals for accommodation) were reversed such that those terms indicated that the eye should accommodate in one direction while defocus indicated that it should accommodate in the other direction. In one of two conditions, two of four observers accommodated initially in the direction specified by the reversed HOAs. Thus, HOAs may be used to drive accommodation in some conditions in some individuals.

#### Diffraction

Diffraction arises from the wave nature of light. When a beam of light passes through an aperture such as the pupil, the beam spreads to create a PSF called the Airy pattern. The intense region in the center of the pattern is the Airy disk, which has a diameter of

\newcommand{\bialpha}{\boldsymbol{\alpha}}\newcommand{\bibeta}{\boldsymbol{\beta}}\newcommand{\bigamma}{\boldsymbol{\gamma}}\newcommand{\bidelta}{\boldsymbol{\delta}}\newcommand{\bivarepsilon}{\boldsymbol{\varepsilon}}\newcommand{\bizeta}{\boldsymbol{\zeta}}\newcommand{\bieta}{\boldsymbol{\eta}}\newcommand{\bitheta}{\boldsymbol{\theta}}\newcommand{\biiota}{\boldsymbol{\iota}}\newcommand{\bikappa}{\boldsymbol{\kappa}}\newcommand{\bilambda}{\boldsymbol{\lambda}}\newcommand{\bimu}{\boldsymbol{\mu}}\newcommand{\binu}{\boldsymbol{\nu}}\newcommand{\bixi}{\boldsymbol{\xi}}\newcommand{\biomicron}{\boldsymbol{\micron}}\newcommand{\bipi}{\boldsymbol{\pi}}\newcommand{\birho}{\boldsymbol{\rho}}\newcommand{\bisigma}{\boldsymbol{\sigma}}\newcommand{\bitau}{\boldsymbol{\tau}}\newcommand{\biupsilon}{\boldsymbol{\upsilon}}\newcommand{\biphi}{\boldsymbol{\phi}}\newcommand{\bichi}{\boldsymbol{\chi}}\newcommand{\bipsi}{\boldsymbol{\psi}}\newcommand{\biomega}{\boldsymbol{\omega}}\begin{equation}\tag{2}\beta = {{2.44\lambda } \over A}\end{equation}

where again *β* is the diameter in radians, *λ* is wavelength in meters, and *A* is pupil diameter in meters. The effect of diffraction is small except at small pupil diameters, which only occur under very bright illumination.

### Micro-fluctuations

Micro-fluctuations (MFs) are involuntary variations in focal power of ∼0.25–0.5 D (Collins, 1939; Arnulf, Dupuy, & Flamant, 1951; Winn & Gilmartin, 1992; Charman & Heron, 1988, 2015). There are high- and low-frequency variations at 1–2 Hz and ∼0.6 Hz, respectively. The consensus is that high frequencies are driven by cardiopulmonary responses while low frequencies are driven by ciliary muscle action on the lens (Winn, Pugh, Gilmartin, & Owens, 1990; Collins, Davis, & Wood, 1995; Walsh & Charman, 1988). MFs could create a directional signal for accommodation (Alpern, 1958; Campbell, Robson, & Westheimer, 1959; Charman & Tucker, 1978; Kotulak & Schor, 1986; Walsh & Charman, 1988). If the eye were out of focus, a fluctuation in one direction would sharpen the retinal image, while a fluctuation in the other direction would blur it further. From the difference, the direction the eye should respond could be determined. Some of the earliest adaptive-optics systems for imaging through the atmosphere used exactly this principle, which is known as multidither (O'Meara, 1977).

There have been hundreds of investigations of MFs (Gray, Winn, & Gilmartin, 1993a,Stark & Atchison, 1997; Gray, Winn, & Gilmartin, 1993b; Denieul & Corno-Martin, 1994; Niwa & Tokoro, 1998; Day, Gray, Seidel, & Strang, 2009; Toshida, Okuyama, & Tokoro, 1998; Heron & Schor, 1995; Seidel, Gray, & Heron, 2005; Harb, Thorn, & Troilo, 2006; Langaas et al., 2008; Winn et al., 1990). Despite this mass of data, we are aware of no studies that directly tested whether MFs are actually used in accommodative control. The decisive experiment has not been done because proper manipulation of MFs is technically quite challenging.

### Chromatic aberration

**Figure 2:**
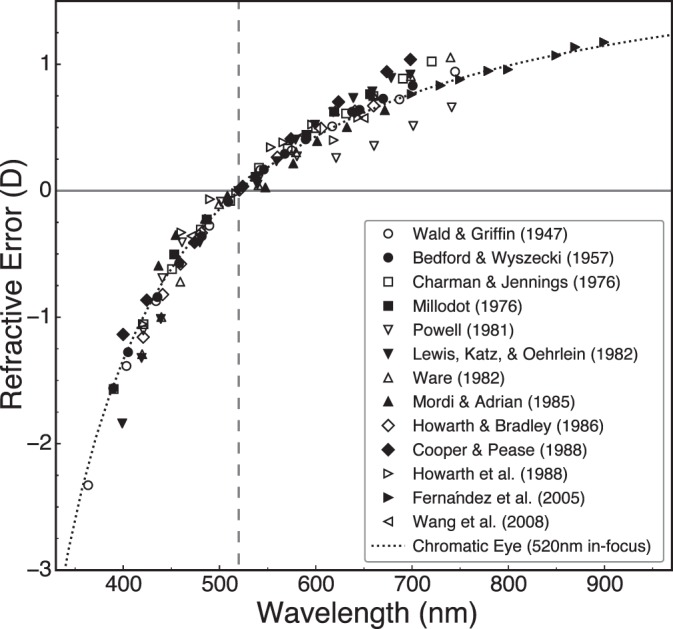
Longitudinal chromatic aberration of the human eye plotted as relative defocus in diopters as a function of wavelength. The dotted curve is the aberration of the chromatic eye model of Thibos et al. (1992). The data were adjusted such that defocus is zero at 520 nm (dashed vertical line); see Equation 3. Data have been replotted from Wald and Griffin (1947), Bedford and Wyszecki (1957), Charman and Jennings (1976), Millodot (1976), Powell (1981), Lewis, Katz, and Oehrlein (1982), Ware (1982), Mordi and Adrian (1985), Howarth and Bradley (1986), Cooper and Pease (1988), Howarth, Zhang, Bradley, Still, & Thibos (1988), Fernández et al. (2005), and Wang, Candy, Teel, & Jacobs (2008).

The eye's refracting elements have different refractive indices for different wavelengths yielding chromatic aberration. As shown in Figure 2, short wavelengths (e.g., blue) are refracted more than long wavelengths (red), so blue and red images tend to be focused respectively in front of and behind the retina. The wavelength-dependent difference in focal distance is longitudinal chromatic aberration (LCA). In diopters:

\newcommand{\bialpha}{\boldsymbol{\alpha}}\newcommand{\bibeta}{\boldsymbol{\beta}}\newcommand{\bigamma}{\boldsymbol{\gamma}}\newcommand{\bidelta}{\boldsymbol{\delta}}\newcommand{\bivarepsilon}{\boldsymbol{\varepsilon}}\newcommand{\bizeta}{\boldsymbol{\zeta}}\newcommand{\bieta}{\boldsymbol{\eta}}\newcommand{\bitheta}{\boldsymbol{\theta}}\newcommand{\biiota}{\boldsymbol{\iota}}\newcommand{\bikappa}{\boldsymbol{\kappa}}\newcommand{\bilambda}{\boldsymbol{\lambda}}\newcommand{\bimu}{\boldsymbol{\mu}}\newcommand{\binu}{\boldsymbol{\nu}}\newcommand{\bixi}{\boldsymbol{\xi}}\newcommand{\biomicron}{\boldsymbol{\micron}}\newcommand{\bipi}{\boldsymbol{\pi}}\newcommand{\birho}{\boldsymbol{\rho}}\newcommand{\bisigma}{\boldsymbol{\sigma}}\newcommand{\bitau}{\boldsymbol{\tau}}\newcommand{\biupsilon}{\boldsymbol{\upsilon}}\newcommand{\biphi}{\boldsymbol{\phi}}\newcommand{\bichi}{\boldsymbol{\chi}}\newcommand{\bipsi}{\boldsymbol{\psi}}\newcommand{\biomega}{\boldsymbol{\omega}}\begin{equation}\tag{3}D(\lambda ) = 2.071 - {{633.46} \over {\lambda - 214.10}}\end{equation}

where *λ* is in nanometers and 520 nm is in-focus (Marimont & Wandell, 1994). From 400–700 nm, the difference is ∼2.5 D. LCA is essentially the same in all human eyes (Nakajima, Hiraoka, Hirohara, Oshika, & Mihashi, 2015; Thibos et al., 1992).

When the eye views a depth-varying scene, LCA produces different color effects (e.g., colored fringes) for different object distances relative to the current focus distance. For example, when the eye is focused on a white point, green is sharp in the retinal image and red and blue are not, so a purple fringe is seen around a sharp greenish center. But when the eye is focused nearer than the white point, the image has a sharp red center surrounded by a blue-green fringe. For far focus, the opposite occurs (Figure 1). Thus, LCA can in principle indicate whether the eye is well focused and, if it is not, in which direction it should accommodate to restore sharp focus.

The eye's chromatic aberration also produces lateral effects in which different wavelengths are imaged at different positions on the retina (Thibos, Bradley, Still, Zhang, & Howarth, 1990). This transverse chromatic aberration is not depth-dependent like LCA and therefore provides no obvious signal to guide accommodation.

The color effects associated with LCA are generally not perceived, but there is clear evidence that they affect accommodation. In a series of persuasive experiments, Kruger and colleagues presented stimuli of constant retinal size to one eye and measured responses to sinusoidal changes in focal distance (Aggarwala, Kruger, Mathews, & Kruger, 1995; Kruger et al., 1993; Stone et al., 1993). Using special lenses, they either eliminated LCA (blue, green, and red images formed at the same distance relative to the retina), reversed it (blue and red images formed behind and in front of the retina, respectively), or left it unaltered. Accommodation was accurate when LCA was unaltered and significantly less accurate when LCA was nulled or reversed. There is also evidence that LCA affects depth perception. Zannoli, Love, Narain, and Banks (2016) briefly presented two broadband abutting surfaces monocularly at different focal distances. Subjects perceived depth order correctly. But when the wavelength spectrum of the stimulus was made narrower (making the LCA signal less useful), performance declined significantly. See also Nguyen, Howard, and Allison (2005). These accommodation and depth perception results are good evidence that LCA contributes to visual function even though the resulting color fringes are not necessarily consciously perceived. With appropriate depth-dependent rendering of color (Cholewiak et al., 2017), one may be able to drive accommodation quite effectively.

## Rendering method

### Calculating retinal images

Our aim is to create displayed images that when viewed by a typical human eye, with its optical imperfections, will produce images on the retina that are the same as those produced by viewing real scenes. In an earlier paper, we describe how to do this for complex 3D scenes using computer graphics (Cholewiak et al., 2017). Here we describe the general problem of which blur kernels are most appropriate for creating realistic retinal images. And then we describe a method for implementing realistic blur for 2 D scenes (i.e., fronto-parallel surfaces at different distances).

In vision science, defocus is simulated in various ways, but by far the most common approach is to convolve parts of the scene with 2 D Gaussians (Mather & Smith, 2002; Watson & Ahumada, 2011; Duchowski et al., 2014; Subedar & Karam, 2016). In computer graphics, ray tracing is used to create depth-dependent blur in complex scenes (Cook, Porter, & Carpenter, 1984). For nondepth-varying scenes, to which we restrict ourselves in this paper, this is equivalent to convolving the scene with a cylinder function whose diameter is given by Equation 1.

But Gaussian and cylinder blur kernels are not equivalent to the PSFs in real optical systems like the eye. According to wave optics for incoherent imaging (Goodman, 1996), the PSF at one wavelength,$\newcommand{\bialpha}{\boldsymbol{\alpha}}\newcommand{\bibeta}{\boldsymbol{\beta}}\newcommand{\bigamma}{\boldsymbol{\gamma}}\newcommand{\bidelta}{\boldsymbol{\delta}}\newcommand{\bivarepsilon}{\boldsymbol{\varepsilon}}\newcommand{\bizeta}{\boldsymbol{\zeta}}\newcommand{\bieta}{\boldsymbol{\eta}}\newcommand{\bitheta}{\boldsymbol{\theta}}\newcommand{\biiota}{\boldsymbol{\iota}}\newcommand{\bikappa}{\boldsymbol{\kappa}}\newcommand{\bilambda}{\boldsymbol{\lambda}}\newcommand{\bimu}{\boldsymbol{\mu}}\newcommand{\binu}{\boldsymbol{\nu}}\newcommand{\bixi}{\boldsymbol{\xi}}\newcommand{\biomicron}{\boldsymbol{\micron}}\newcommand{\bipi}{\boldsymbol{\pi}}\newcommand{\birho}{\boldsymbol{\rho}}\newcommand{\bisigma}{\boldsymbol{\sigma}}\newcommand{\bitau}{\boldsymbol{\tau}}\newcommand{\biupsilon}{\boldsymbol{\upsilon}}\newcommand{\biphi}{\boldsymbol{\phi}}\newcommand{\bichi}{\boldsymbol{\chi}}\newcommand{\bipsi}{\boldsymbol{\psi}}\newcommand{\biomega}{\boldsymbol{\omega}}PS{F_\lambda }(\theta ,\phi )$, is proportional to the square modulus of the Fourier transform of the complex aperture function, which takes into account both the amplitude and phase of the input:

\newcommand{\bialpha}{\boldsymbol{\alpha}}\newcommand{\bibeta}{\boldsymbol{\beta}}\newcommand{\bigamma}{\boldsymbol{\gamma}}\newcommand{\bidelta}{\boldsymbol{\delta}}\newcommand{\bivarepsilon}{\boldsymbol{\varepsilon}}\newcommand{\bizeta}{\boldsymbol{\zeta}}\newcommand{\bieta}{\boldsymbol{\eta}}\newcommand{\bitheta}{\boldsymbol{\theta}}\newcommand{\biiota}{\boldsymbol{\iota}}\newcommand{\bikappa}{\boldsymbol{\kappa}}\newcommand{\bilambda}{\boldsymbol{\lambda}}\newcommand{\bimu}{\boldsymbol{\mu}}\newcommand{\binu}{\boldsymbol{\nu}}\newcommand{\bixi}{\boldsymbol{\xi}}\newcommand{\biomicron}{\boldsymbol{\micron}}\newcommand{\bipi}{\boldsymbol{\pi}}\newcommand{\birho}{\boldsymbol{\rho}}\newcommand{\bisigma}{\boldsymbol{\sigma}}\newcommand{\bitau}{\boldsymbol{\tau}}\newcommand{\biupsilon}{\boldsymbol{\upsilon}}\newcommand{\biphi}{\boldsymbol{\phi}}\newcommand{\bichi}{\boldsymbol{\chi}}\newcommand{\bipsi}{\boldsymbol{\psi}}\newcommand{\biomega}{\boldsymbol{\omega}}\begin{equation}\tag{4}PS{F_\lambda }(\theta ,\phi ) \propto {\left| {\mathfrak{\cal {F}}\left( {A{e^{{{2\pi i} \over \lambda }\left( {{Z_d} + {Z_{HOA}} + {Z_{LCA}}} \right)}}} \right)} \right|^2}\end{equation}

where *θ* and *ϕ* are the angular coordinates on the retina, *λ* is again wavelength (we assume for now that all are transmitted equally; a term can be added to account for wavelength dependence), $\newcommand{\bialpha}{\boldsymbol{\alpha}}\newcommand{\bibeta}{\boldsymbol{\beta}}\newcommand{\bigamma}{\boldsymbol{\gamma}}\newcommand{\bidelta}{\boldsymbol{\delta}}\newcommand{\bivarepsilon}{\boldsymbol{\varepsilon}}\newcommand{\bizeta}{\boldsymbol{\zeta}}\newcommand{\bieta}{\boldsymbol{\eta}}\newcommand{\bitheta}{\boldsymbol{\theta}}\newcommand{\biiota}{\boldsymbol{\iota}}\newcommand{\bikappa}{\boldsymbol{\kappa}}\newcommand{\bilambda}{\boldsymbol{\lambda}}\newcommand{\bimu}{\boldsymbol{\mu}}\newcommand{\binu}{\boldsymbol{\nu}}\newcommand{\bixi}{\boldsymbol{\xi}}\newcommand{\biomicron}{\boldsymbol{\micron}}\newcommand{\bipi}{\boldsymbol{\pi}}\newcommand{\birho}{\boldsymbol{\rho}}\newcommand{\bisigma}{\boldsymbol{\sigma}}\newcommand{\bitau}{\boldsymbol{\tau}}\newcommand{\biupsilon}{\boldsymbol{\upsilon}}\newcommand{\biphi}{\boldsymbol{\phi}}\newcommand{\bichi}{\boldsymbol{\chi}}\newcommand{\bipsi}{\boldsymbol{\psi}}\newcommand{\biomega}{\boldsymbol{\omega}}\mathfrak{\cal {F}}$ denotes the Fourier transform, *A* describes the transmittance of the pupil (assumed to be a cylinder function) as a function of coordinates *x* and *y*, and *Z* defines the wavefront aberration in the eye also as a function of *x* and *y*. We separate the function into terms for defocus (*d*), higher-order aberrations (*HOA*, which includes astigmatism), and longitudinal chromatic aberration (*LCA*). Once the PSF is known, the retinal image can be calculated by convolving it with the scene (assuming no depth variation in the scene and a field of view small enough to justify the assumption of a constant PSF over the field; Navarro, Williams, & Artal, 1993). We refer to this calculated image as the target retinal image. For an in-focus eye with no aberrations, the PSF is the Airy pattern.

**Figure 3:**
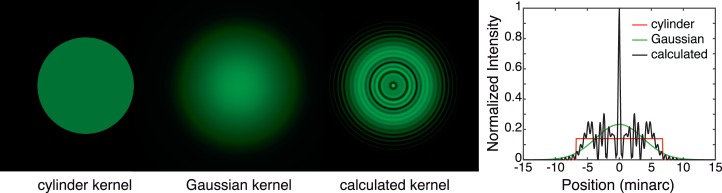
Left: Three blur kernels for rendering an image with 1 D of defocus for a 4 mm pupil and 550 nm. From left to right, they are a cylinder, Gaussian, and a function calculated from Equation 4 and described in the next section. The standard deviation of the Gaussian is 0.55 times the radius of the cylinder. The functions have been normalized to have the same volumes. Right: Cross-sections of these functions. Red, green, and black represent respectively the cylinder function, Gaussian function, and the function calculated from Equation 4, again normalized to equal volume.

**Figure 4:**
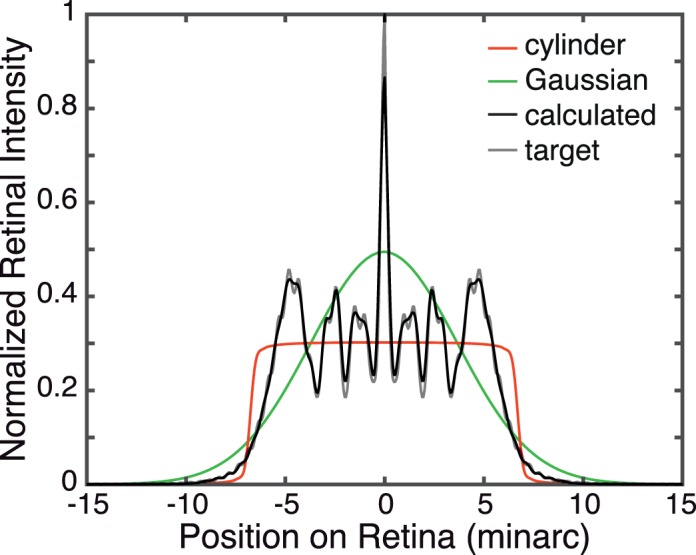
Cross sections of PSFs at the retina resulting from viewing blur kernels in Figure 3. The viewing situation is the same as in that figure: 4 mm pupil, 1 D of defocus, 550 nm. Red represents the retinal PSF when a cylinder function is used for the blur kernel at the display. Green is the PSF when a Gaussian function is used at the display. Gray is the target retinal PSF. Black represents the retinal PSF when the display blur kernel is produced by Equation 8. The gray and black lines are nearly identical, which illustrates that our rendered PSF, viewed through the optics of the eye, produces a retinal image very close to the target image.

**Figure 5:**
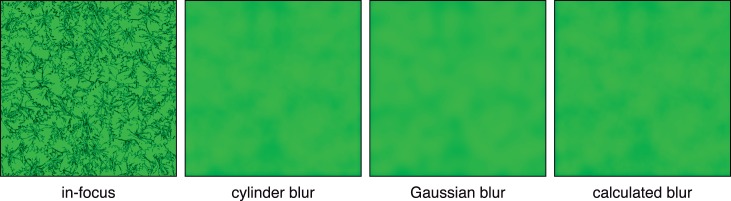
Displayed images produced using the blur kernels in Figure 3: 4 mm pupil, 1 D of defocus, 550 nm. Left: Sharp, in-focus image. Middle-left: Displayed image created by blurring the left image with the cylinder function. Middle-right: Gaussian blur. Right: calculated blur kernel from Equation 4. For the correct scale, view the images at a distance of 28 times the height of individual panels. Ideally, pupil diameter should be 4 mm. The differences are subtle when printed, but are more apparent if you examine on a display screen from a closer distance.

The PSF is more complex for a defocused or aberrated eye, which is illustrated in Figures 1 and 3. The left panel of Figure 3 shows, from left to right, cylinder, Gaussian, and calculated blur kernels for a 4 mm pupil, 1 D of defocus, and monochromatic light of 550 nm. The cylinder kernel is derived from Equation 1. The Gaussian kernel has a standard deviation of 0.55 times the radius of the cylinder; that value minimizes RMS error between the two functions. The calculated kernel is derived from Equation 4; the procedure involved in generating it is described in the next section. The right panel shows cross-sections through these kernels. Figure 4 shows the retinal PSFs that result from an in-focus eye viewing the blur kernels in Figure 3. The thin gray line represents the target PSF and the black line the one produced by our method (which is described in the following section); the agreement between the two is excellent. Figure 5 shows the blurred displayed images produced by the three kernels. The images when viewed from the correct distance (see caption) differ subtly. The Gaussian kernel degrades the image the most because that PSF is smooth, which attenuates high spatial frequencies more than the other kernels. In a forthcoming paper, we explore how the type of kernel used affects accommodation and perceived depth (Cholewiak, Shirley, McGuire, & Banks, 2018).

### Calculating displayed images

Again we want to find the displayed image that when viewed by the imperfect eye will produce a retinal image as similar as possible to the target retinal image. If the scene is meant to appear in focus, the solution is straightforward: One generates a displayed image of high quality (i.e., no defocus or aberrations). When that image is viewed by an in-focus eye, the effects of diffraction, HOAs, and LCA are inserted by the viewer's eye, and the correct retinal image is produced. The solution is much less straightforward for creating displayed images that are meant to appear out of focus (or distorted by another aberration), but are viewed by an in-focus eye. We can calculate the desired retinal image using Equation 4, but to create the correct displayed image, we must account for the effects introduced by the viewer's eye.

#### Deconvolution

Although we do not use deconvolution in this paper, it is useful to discuss it in order to illuminate problems that arise in calculating correct displayed images. The input image is *I_input_* and the PSF needed to render the displayed image *I_display_* is *PSF_display_*. We first calculate via Equation 4 the in- and out-of-focus PSFs at the retina: *PSF_infocus_* and *PSF_defocus_*, respectively. The target retinal image that would be produced in the real world is

\newcommand{\bialpha}{\boldsymbol{\alpha}}\newcommand{\bibeta}{\boldsymbol{\beta}}\newcommand{\bigamma}{\boldsymbol{\gamma}}\newcommand{\bidelta}{\boldsymbol{\delta}}\newcommand{\bivarepsilon}{\boldsymbol{\varepsilon}}\newcommand{\bizeta}{\boldsymbol{\zeta}}\newcommand{\bieta}{\boldsymbol{\eta}}\newcommand{\bitheta}{\boldsymbol{\theta}}\newcommand{\biiota}{\boldsymbol{\iota}}\newcommand{\bikappa}{\boldsymbol{\kappa}}\newcommand{\bilambda}{\boldsymbol{\lambda}}\newcommand{\bimu}{\boldsymbol{\mu}}\newcommand{\binu}{\boldsymbol{\nu}}\newcommand{\bixi}{\boldsymbol{\xi}}\newcommand{\biomicron}{\boldsymbol{\micron}}\newcommand{\bipi}{\boldsymbol{\pi}}\newcommand{\birho}{\boldsymbol{\rho}}\newcommand{\bisigma}{\boldsymbol{\sigma}}\newcommand{\bitau}{\boldsymbol{\tau}}\newcommand{\biupsilon}{\boldsymbol{\upsilon}}\newcommand{\biphi}{\boldsymbol{\phi}}\newcommand{\bichi}{\boldsymbol{\chi}}\newcommand{\bipsi}{\boldsymbol{\psi}}\newcommand{\biomega}{\boldsymbol{\omega}}\begin{equation}\tag{5}{I_{retina}} = {I_{input}} \circledast PS{F_{defocus}}\end{equation}

where $\newcommand{\bialpha}{\boldsymbol{\alpha}}\newcommand{\bibeta}{\boldsymbol{\beta}}\newcommand{\bigamma}{\boldsymbol{\gamma}}\newcommand{\bidelta}{\boldsymbol{\delta}}\newcommand{\bivarepsilon}{\boldsymbol{\varepsilon}}\newcommand{\bizeta}{\boldsymbol{\zeta}}\newcommand{\bieta}{\boldsymbol{\eta}}\newcommand{\bitheta}{\boldsymbol{\theta}}\newcommand{\biiota}{\boldsymbol{\iota}}\newcommand{\bikappa}{\boldsymbol{\kappa}}\newcommand{\bilambda}{\boldsymbol{\lambda}}\newcommand{\bimu}{\boldsymbol{\mu}}\newcommand{\binu}{\boldsymbol{\nu}}\newcommand{\bixi}{\boldsymbol{\xi}}\newcommand{\biomicron}{\boldsymbol{\micron}}\newcommand{\bipi}{\boldsymbol{\pi}}\newcommand{\birho}{\boldsymbol{\rho}}\newcommand{\bisigma}{\boldsymbol{\sigma}}\newcommand{\bitau}{\boldsymbol{\tau}}\newcommand{\biupsilon}{\boldsymbol{\upsilon}}\newcommand{\biphi}{\boldsymbol{\phi}}\newcommand{\bichi}{\boldsymbol{\chi}}\newcommand{\bipsi}{\boldsymbol{\psi}}\newcommand{\biomega}{\boldsymbol{\omega}}\circledast$ denotes convolution. To reproduce that retinal image, the image to be displayed must take into account the contribution of the viewer's in-focus optics. We want

\newcommand{\bialpha}{\boldsymbol{\alpha}}\newcommand{\bibeta}{\boldsymbol{\beta}}\newcommand{\bigamma}{\boldsymbol{\gamma}}\newcommand{\bidelta}{\boldsymbol{\delta}}\newcommand{\bivarepsilon}{\boldsymbol{\varepsilon}}\newcommand{\bizeta}{\boldsymbol{\zeta}}\newcommand{\bieta}{\boldsymbol{\eta}}\newcommand{\bitheta}{\boldsymbol{\theta}}\newcommand{\biiota}{\boldsymbol{\iota}}\newcommand{\bikappa}{\boldsymbol{\kappa}}\newcommand{\bilambda}{\boldsymbol{\lambda}}\newcommand{\bimu}{\boldsymbol{\mu}}\newcommand{\binu}{\boldsymbol{\nu}}\newcommand{\bixi}{\boldsymbol{\xi}}\newcommand{\biomicron}{\boldsymbol{\micron}}\newcommand{\bipi}{\boldsymbol{\pi}}\newcommand{\birho}{\boldsymbol{\rho}}\newcommand{\bisigma}{\boldsymbol{\sigma}}\newcommand{\bitau}{\boldsymbol{\tau}}\newcommand{\biupsilon}{\boldsymbol{\upsilon}}\newcommand{\biphi}{\boldsymbol{\phi}}\newcommand{\bichi}{\boldsymbol{\chi}}\newcommand{\bipsi}{\boldsymbol{\psi}}\newcommand{\biomega}{\boldsymbol{\omega}}\begin{equation}\tag{6}{I_{retina}} = \left( {{I_{input}} \circledast PS{F_{display}}} \right) \circledast PS{F_{infocus}}\end{equation}

Combining the equations and rearranging:

\newcommand{\bialpha}{\boldsymbol{\alpha}}\newcommand{\bibeta}{\boldsymbol{\beta}}\newcommand{\bigamma}{\boldsymbol{\gamma}}\newcommand{\bidelta}{\boldsymbol{\delta}}\newcommand{\bivarepsilon}{\boldsymbol{\varepsilon}}\newcommand{\bizeta}{\boldsymbol{\zeta}}\newcommand{\bieta}{\boldsymbol{\eta}}\newcommand{\bitheta}{\boldsymbol{\theta}}\newcommand{\biiota}{\boldsymbol{\iota}}\newcommand{\bikappa}{\boldsymbol{\kappa}}\newcommand{\bilambda}{\boldsymbol{\lambda}}\newcommand{\bimu}{\boldsymbol{\mu}}\newcommand{\binu}{\boldsymbol{\nu}}\newcommand{\bixi}{\boldsymbol{\xi}}\newcommand{\biomicron}{\boldsymbol{\micron}}\newcommand{\bipi}{\boldsymbol{\pi}}\newcommand{\birho}{\boldsymbol{\rho}}\newcommand{\bisigma}{\boldsymbol{\sigma}}\newcommand{\bitau}{\boldsymbol{\tau}}\newcommand{\biupsilon}{\boldsymbol{\upsilon}}\newcommand{\biphi}{\boldsymbol{\phi}}\newcommand{\bichi}{\boldsymbol{\chi}}\newcommand{\bipsi}{\boldsymbol{\psi}}\newcommand{\biomega}{\boldsymbol{\omega}}\begin{equation}\tag{7}PS{F_{defocus}} = PS{F_{display}} \circledast PS{F_{infocus}}\end{equation}

Transforming into the Fourier domain:

\newcommand{\bialpha}{\boldsymbol{\alpha}}\newcommand{\bibeta}{\boldsymbol{\beta}}\newcommand{\bigamma}{\boldsymbol{\gamma}}\newcommand{\bidelta}{\boldsymbol{\delta}}\newcommand{\bivarepsilon}{\boldsymbol{\varepsilon}}\newcommand{\bizeta}{\boldsymbol{\zeta}}\newcommand{\bieta}{\boldsymbol{\eta}}\newcommand{\bitheta}{\boldsymbol{\theta}}\newcommand{\biiota}{\boldsymbol{\iota}}\newcommand{\bikappa}{\boldsymbol{\kappa}}\newcommand{\bilambda}{\boldsymbol{\lambda}}\newcommand{\bimu}{\boldsymbol{\mu}}\newcommand{\binu}{\boldsymbol{\nu}}\newcommand{\bixi}{\boldsymbol{\xi}}\newcommand{\biomicron}{\boldsymbol{\micron}}\newcommand{\bipi}{\boldsymbol{\pi}}\newcommand{\birho}{\boldsymbol{\rho}}\newcommand{\bisigma}{\boldsymbol{\sigma}}\newcommand{\bitau}{\boldsymbol{\tau}}\newcommand{\biupsilon}{\boldsymbol{\upsilon}}\newcommand{\biphi}{\boldsymbol{\phi}}\newcommand{\bichi}{\boldsymbol{\chi}}\newcommand{\bipsi}{\boldsymbol{\psi}}\newcommand{\biomega}{\boldsymbol{\omega}}\begin{equation}\tag{8}OT{F_{display}} = {{OT{F_{defocus}}} \over {OT{F_{infocus}}}}\end{equation}

where *OTF* is the optical transfer function. As you can see, *OTF_display_* is undefined for spatial frequencies at which *OTF_infocus_* is zero. Furthermore, the displayed image may be unrealizable when *OTF_infocus_* has small values because the display would need to have contrasts greater than 1.

#### Search algorithm

Many applications of deconvolution involve recovering a signal from data corrupted by an unknown process (e.g., atmospheric aberrations) in the presence of noise. In our case, there is no noise and the aberrations are known. We take advantage by using an alternative procedure that avoids some problems in deconvolution. Here we describe the procedure when the eye model has diffraction, defocus, and LCA. The procedure is quite similar when incorporating other aberrations such as astigmatism.

We first solve the forward problem of calculating the retinal image that should be produced given the scene and parameters of the eye model. This produces the target retinal image (Equation 5). We then search across various defocus values for the red, green, and blue primaries to find the displayed image that when processed by the viewer's in-focus eye produces a retinal image that best matches the target (minimizing RMS error).

**Figure 6:**
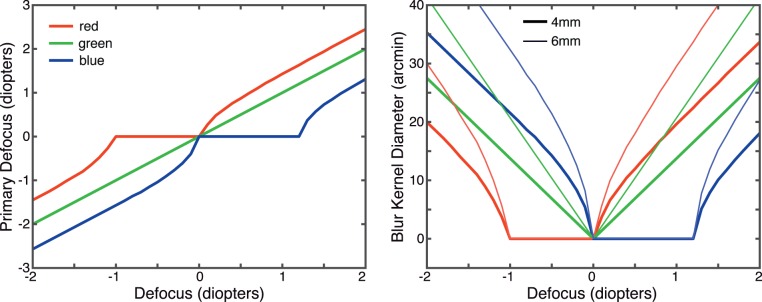
Defocus and blur kernels for rendering. Left: The defocus values for each primary as a function of simulated defocus value. The viewer's eye is focused at 520 nm. The red, green, and blue lines represent the defocus values for each primary that one should use when simulating different object distances. The wavelengths for the R, G, and B primaries are assumed to be 617, 520, and 449 nm, respectively. Right: The diameters of cylindrical blur kernels to use in rendering for R, G, and B primaries. Pupil diameter is 4 mm (thick lines) and 6 mm (thin lines). The values were calculated from the left panel and Equation 1. These are not the kernels we used to generate the stimuli because they included diffraction effects as shown in Figures 3 and 4.

Figure 6 shows results. The left panel shows the best defocus values to render an image for three color primaries (449, 520, and 617 nm, the peak wavelengths of our projector's emission spectra). We assume the eye is focused at 520 nm and show the defocus values that, when propagated through the in-focus eye, produce the best match to the target retinal images. The function for G is linear because we assume the viewing eye is in focus at that wavelength, so his/her LCA does not contribute. The functions for R and B are roughly linear at large simulated defocus values because at those values the main determinant of blur in the retinal image is the dioptric distance of the object relative to the simulated focus distance of the eye. In other words, at large defocus values, the required values for R and B are approximately proportional to the defocus of the object we wish to simulate. The functions deviate from such proportionality at small simulated defocus values because the main determinant of retinal blur at those values is the LCA of the viewer's eye.

The right panel of Figure 6 shows the diameters of the blur kernels one should use to create the closest match to the correct retinal image. The functions terminate at an ordinate value of 0 because the kernels cannot have negative width. For simplicity, we use cylindrical kernels here, which are derived from the defocus values on the left and Equation 1. In our experiments we used more complex kernels derived from Equation 4 and shown in Figure 4. These results are based on an eye model with defocus and LCA only. We examined how incorporating other aberrations affects these calculations and found that the effects are quite small in most eyes (Supplementary Figure S2).

Our rendering method, which incorporates LCA, is much more accurate than conventional methods, which do not (Cholewiak et al., 2017; Figures 4 through 6). Our method becomes less accurate (but still more accurate than conventional methods) at small defocus values (Cholewiak et al., 2017).

**Figure 7:**
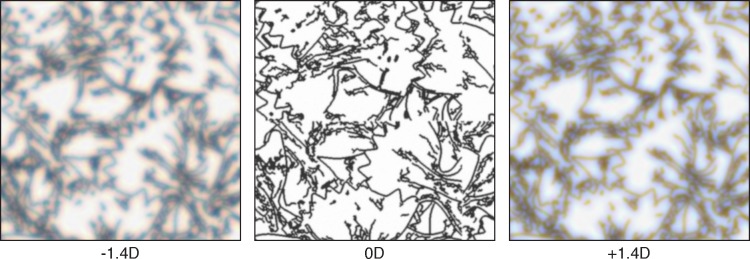
Example stimuli generated using our rendering method. The left, middle, and right images are simulated to be farther, at, and nearer than current focus. The panels should be viewed with a pupil of ∼6 mm from a distance of 5.6 times the height of the individual panels. Ideally, the spectra of the display would be similar to the ones used in our experiments. See Supplementary Material for information about the display spectra (Supplementary Figure S1 and Supplementary Table S1) and for more examples (Supplementary Figure S3).

Figure 7 provides examples of the resulting displayed images: one for -1.4 D of defocus, one for 0 D, and one for +1.4 D.

## Longitudinal chromatic aberration

We first examined how incorporation of LCA in blur rendering affects accommodation. We compared accommodative responses to real changes in focal distance, simulated changes with each color treated in the same fashion, and simulated changes with each color treated in an appropriate depth-dependent fashion.

Previously we observed that our rendering method does in fact stimulate accommodative responses to step changes in simulated focal distance (Cholewiak et al., 2017). We also observed no consistent responses to simulated step changes with conventional blur rendering.

When the focal distance of a visual stimulus is oscillated sinusoidally in time such that all blur cues (e.g., defocus, LCA, spherical aberration, etc.) are consistent with one another, accommodation oscillates at the same frequency. Response amplitude is similar to stimulus amplitude at frequencies up to 0.2 Hz, but decreases at higher frequencies until no response is observed at 2–3 Hz (Campbell & Westheimer, 1960; Krishnan, Phillips, & Stark, 1973). There is a time lag between the stimulus and response of 300–400 ms (Campbell & Westheimer, 1960; Krishnan et al., 1973). We wanted to know how accommodative dynamics are affected by simulated changes in focal distance relative to real changes. We measured response gain and phase at a variety of temporal frequencies for real and simulated changes in focal distance. The simulated changes were generated by conventional rendering and by our color-correct rendering method.

### Methods

#### Subjects

Ten naïve subjects (22–26 years old) participated. Nine were female, and one was male. Nine were myopic and wore their prescribed correction during the experiment. One did not require correction. All had normal visual acuity when corrected. Median pupil diameters were 3.6–5.8 mm with an average of 4.7 mm. The experiment was approved by the institutional review board at UC Berkeley and conducted in accordance with the Declaration of Helsinki.

#### Apparatus

**Figure 8:**
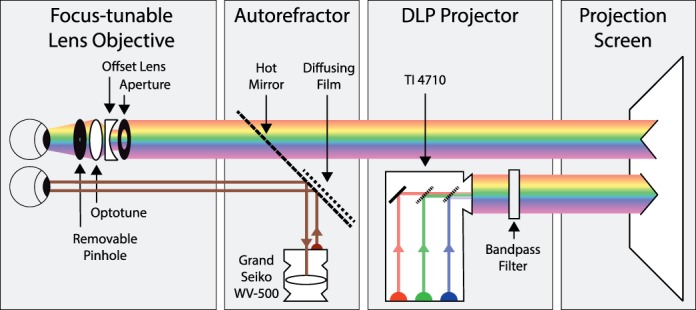
Schematic of experimental apparatus. The DLP projector delivered images to the projection screen through a Chroma triple-bandpass filter. The color primaries were three LEDs. The subject viewed the stimulus on the projection screen with their left eye. A focus-adjustable lens, fixed offset lens, and aperture were placed just in front of that eye. The subject viewed the stimuli through the lenses and aperture. For some experimental conditions, a pinhole was placed near the cornea. An autorefractor delivered infrared light to the right eye. The light was reflected by a hot mirror that reflects infrared but transmits visible light. The reflection of that light from the right eye's retina was recorded by the autorefractor's video camera.

Figure 8 shows the experimental setup. We stimulated the left eye while measuring accommodation in the right eye. Accommodation is yoked between eyes (Campbell, 1960; Fisher, Ciuffreda, & Hammer, 1987), so this method is well justified. Stimuli were projected onto a screen using a DLP projector with a resolution of 1,920 × 1,080. The R, G, and B primaries were LEDs with relatively narrow spectra. We placed a triple bandpass filter (Chroma 69002 m) in the projector's light path to further narrow the spectra (see Supplementary Material). The projection screen was 1.26 m (0.79 D) from the subject's eye and subtended 32° × 18°. Nyquist frequency was 30 cycles/°. The room was dark except for the projected stimulus.

A focus-adjustable lens (Optotune EL-10-30-TC) was placed just in front of the stimulated eye. We varied the power of this lens to manipulate the focal distance of the stimulus. The lens has nearly zero LCA (Abbe number = 100). We placed a −10 D achromatic lens in the optical path to give a range of potential focal distances at the eye of −3.8 to +10.2 D.

We measured accommodation with an autorefractor (Grand Seiko WV-500). The device projects infrared light into the eye and records the image reflected from the retina. In its normal operating mode, sampling rate is 1 Hz, but the composite video signal provides a much higher rate. Using a method similar to Wolffsohn, O'Donnell, Charman, and Gilmartin (2004) and MacKenzie, Hoffman, and Watt (2010), we were able to measure accommodation at 30 Hz by processing the video offline (see supplementary material of Cholewiak et al. (2017) for more details). We removed data corrupted by eye blinks or eye movements.

#### Stimuli

The experimental stimuli were black-and-white textured fronto-parallel planes. The texture on each trial was chosen randomly from one of four precomputed ones. They had the same space-average luminance and contrast energy, and similar amplitude spectra of ∼1/*f*. The real or simulated focal distance of the textured plane oscillated sinusoidally (in diopters) from 0.5–3.5 D (mean of 2.0 D) at one of seven frequencies (0.05, 0.1, 0.2, 0.4, 0.6, 0.8, or 1.0 Hz).

#### Procedure

Subjects first fixated on and accommodated to a textured plane at 2.0 D with a small black-on-green Maltese-cross fixation target. They were told to fixate and accommodate to keep the target in focus for the duration of trial. After the subject pressed the space bar, the fixation target changed to a black-on-white Maltese cross, and the oscillating stimulus was presented for 10 or 20 s. At the end of the stimulus presentation, the fixation target changed back to the black-on-green cross.

There were three conditions: *Real Change* in which the actual focal distance of the stimulus changed due to changes in the power of the focus-adjustable lens; *Defocus Only* in which the focal distance did not change, but the rendered blur changed by the same amount for all three color primaries (conventional rendering); *Defocus + LCA* in which the focal distance did not change, but the rendered blur changed appropriately for each primary (using our rendering method). Stimulus size at the retina did not vary for any condition.

Conditions and temporal frequencies were presented in random order. In all, 126 trials were presented to each subject: three conditions, seven frequencies, and six repetitions.

It is important to consider the various signals for accommodation in the three conditions. In the *Real Change* condition, all blur signals—defocus, MFs, HOAs, astigmatism, LCA—indicate the same required response. This condition is very similar to the *Optical Blur* condition in Smithline (1974). In the *Defocus Only* condition, the actual focal distance to the stimulus does not change. Defocus specifies that the stimulus distance changed but does not specify the direction of change. The other signals—MFs, HOAs, astigmatism, LCA—all indicate that the eye is well focused and therefore that no response is required. If an accommodative response away from the actual focal distance of the stimulus occurred, defocus would of course increase. This condition is quite similar to the *Rendered Blur* condition in Smithline (1974). The *Defocus + LCA* condition is the same as *Defocus Only* except that LCA indicates the direction and magnitude of the required response; the other signals indicate that the eye is well focused and that no response is needed. If an accommodative response away from the focal distance of the stimulus occurred, defocus would of course increase, indicating that the response was inappropriate for increasing sharpness.

### Results

**Figure 9:**
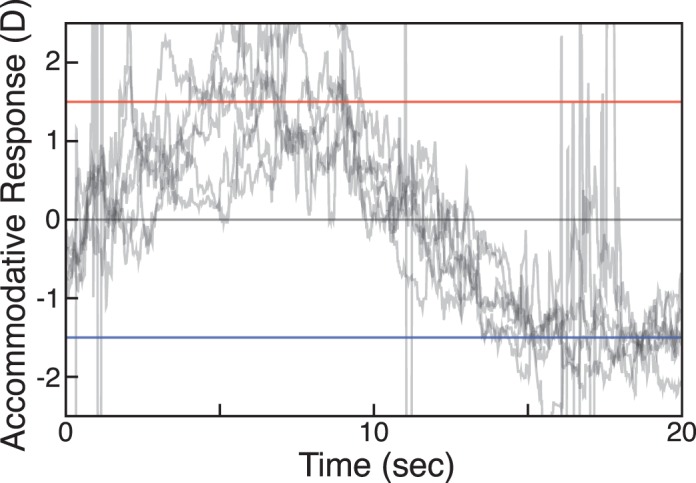
Accommodative responses in the Real Change condition for one subject with 0.05 Hz sinusoidal stimulation. Change in focal distance ranged from +1.5 D (red line) to −1.5 D (blue). Each trace is the response recorded on one trial. Most artifacts due to blinks and eye movements have been removed. The midpoint of the sinusoid was actually 2 D, but here it is plotted as 0 D for convenience.

Figure 9 shows trial-by-trial responses in one condition for a representative subject. The traces are responses on individual trials. They varied somewhat from trial to trial, but were clearly yoked to the stimulus.

**Figure 10:**
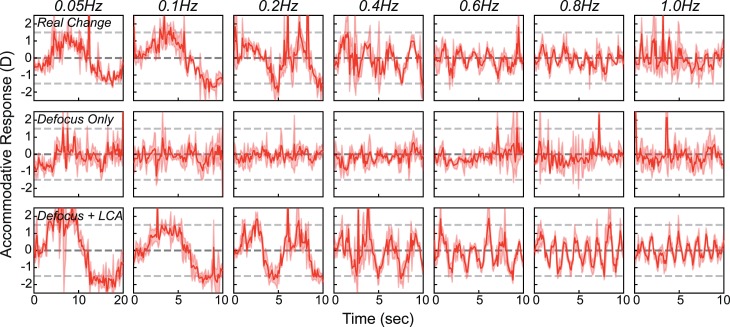
Accommodative responses to sinusoidal changes in real and simulated focal distance. Response in diopters is plotted as a function of time for a stimulus with a peak-to-trough amplitude of 3 D. Data have been smoothed with a running median calculation with a window of 100 ms. Top, middle, and bottom rows show the responses, respectively, for Real Change, Defocus Only, and Defocus + LCA. Columns from left to right show responses for frequencies of 0.05, 0.1, 0.2, 0.4, 0.6, 0.8, and 1.0 Hz. Because we are only interested in differential responses, the DC component (offset in the sinewave fit) was removed from each trace. Thick curves are the median responses. Note the change in scale on the abscissa between the first and second columns.

We subjected each subject's data to a running median calculation with a window of 100 ms, yielding smoothed data like those in Figure 10. (The smoothed data from the other nine subjects are provided in Supplementary Figures S4, S5, and S6.) The rows from top to bottom show the data from the *Real Change*, *Defocus Only*, and *Defocus + LCA* conditions. The columns from left to right show the data at frequencies of 0.05, 0.1, 0.2, 0.4, 0.6, 0.8, and 1.0 Hz, respectively.

Responses in the *Real Change* condition were reasonably accurate at low frequencies and diminished above 0.2 Hz, consistent with previous findings (Campbell & Westheimer, 1960; Krishnan et al., 1973; Kruger & Pola, 1986; Ohtsuka & Sawa, 1997; Van der Wildt, Bouman, & Van de Kraats, 1974). There were no consistent accommodative responses in the *Defocus Only* condition indicating that conventional rendering does not produce reliable responses at any frequency. Interestingly, responses in the *Defocus + LCA* condition were as robust as they were in the *Real Change* condition. Thus, appropriate color and blur rendering was as effective in driving accommodation as real changes were.

We fit the raw data in each condition for each subject with a sinusoid of the same frequency as the driving stimulus. Free parameters were amplitude, phase, and DC offset. We then estimated gains from the ratio of the amplitude of the fit divided by the amplitude of the stimulus. A gain of 1 would indicate that the response amplitude was equal to the stimulus amplitude. A gain of 0 would never be observed with this fitting procedure because the noisy response data will contain some energy at the stimulus frequency even if no stimulus-driven response occurs. We estimated the floor value for the procedure by fitting sinusoids at various stimulus frequencies to response data when no change in real or simulated distance occurred. In that case, the gain estimates were ∼0.05–0.25 depending on temporal frequency, so we regard those values as the floor.

**Figure 11:**
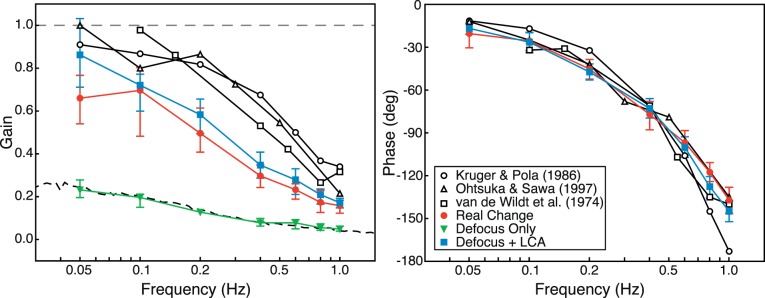
Accommodative gains and phases compared to values from previous experiments. Left: Gain (amplitude of sinewave fit to the response data divided by amplitude of the stimulus) is plotted as a function of frequency. Colored symbols are median gains in our experiment across subjects: red circles for Real Change, green triangles for Defocus Only, and blue squares for Defocus + LCA. The dashed black curve represents expected gains at different frequencies when the stimulus has zero amplitude. Error bars are 95% CI. Black unfilled symbols are data in the real change condition from previous experiments. Right: Phase (phase of the sinewave fit to the data relative to phase of the stimulus) is plotted as a function of frequency. Colored symbols are median phases in our experiment: red circles for Real Change and blue squares for Defocus + LCA. Phase for Defocus Only have been omitted because they are meaningless given the low gain in that condition. Error bars are 95% CI. Black unfilled symbols are data in real change conditions from previous experiments.

The left panel of Figure 11 shows the median gains as a function of temporal frequency for the three conditions. Response gains were very similar in the *Real Change* and *Defocus + LCA* conditions, a result which means that changes in rendered blur with appropriate LCA were as effective at driving accommodation as real changes in focal distance. The gains were very low in the *Defocus Only* condition and very similar to the estimated gains when no driving stimulus was present. The right panel of Figure 11 shows the median phase lags for the *Real Change* and *Defocus + LCA* conditions. (The phase estimates in the *Defocus Only* condition were meaningless because the gains were in effect zero.) The lags in the *Real Change* and *Defocus + LCA* conditions were very similar, again showing that changes in rendered blur with appropriate LCA were very effective in driving accommodation.

We subjected the gain estimates to a repeated-measures ANOVA with factors of subject, condition, and frequency. There were statistically significant effects for all three factors. We conducted multiple pairwise comparisons using Tukey contrasts with Bonferroni correction. The results showed that accommodative gains were significantly greater in the *Real Change* condition than in the *Defocus Only* condition for driving frequencies of 0.05, 0.1, 0.2, 0.4, and 0.6 Hz (i.e., not 0.8 and 1.0 Hz). Accommodation was significantly greater in the *Defocus + LCA* condition than in the *Defocus Only* condition for frequencies of 0.05, 0.1, 0.2, 0.4, 0.6, and 0.8 Hz (i.e., not 1.0 Hz). Gains were greater in the *Defocus + LCA* condition than in the *Real Change* condition at the slowest driving frequency, 0.05 Hz (but not at the other values). Therefore, response gains were consistently greater in the *Real Change* and *Defocus + LCA* conditions than in the *Defocus Only* condition and were generally the same in the *Real Change* and *Defocus + LCA* conditions.

As we noted earlier, an accommodative response in the *Defocus + LCA* condition necessarily causes more blur in the retinal image. Subjects actually noticed this, but nonetheless made responses in the direction specified by *Defocus + LCA*.

This experiment is superficially similar to one by Kruger et al. (1995). In their experiment, the stimulus was a black-white sinewave grating that oscillated sinusoidally in actual or simulated focal distance. They measured gains and phases of the resulting responses. In one condition, the grating stimulus oscillated in actual focal distance as it did in our *Real Change* condition. In the other two conditions, the stimulus oscillated in simulated focal distance. One of the simulated conditions was similar to our *Defocus Only* condition (they called theirs *Luminance Control*). The other was similar to our *Defocus + LCA* condition (theirs was *Chromatic*). In the *Luminance Control* condition, the three color primaries of their stimulus underwent the same changes in contrast, so the depth-dependent effects of LCA were not incorporated. In their *Chromatic* condition, the three primaries underwent different changes in contrast in a fashion consistent with the depth-dependent properties of LCA. In the *Luminance Control* and *Chromatic* conditions, accommodative responses produced no change in the retinal image by virtue of a closed loop in their apparatus. The only retinal-image changes were due to oscillations in the rendered contrast and coloring of the grating. They found that the *Chromatic* condition drove accommodation, but not nearly as effectively as actual changes in focal distance did (see their Figure 12).

**Figure 12:**
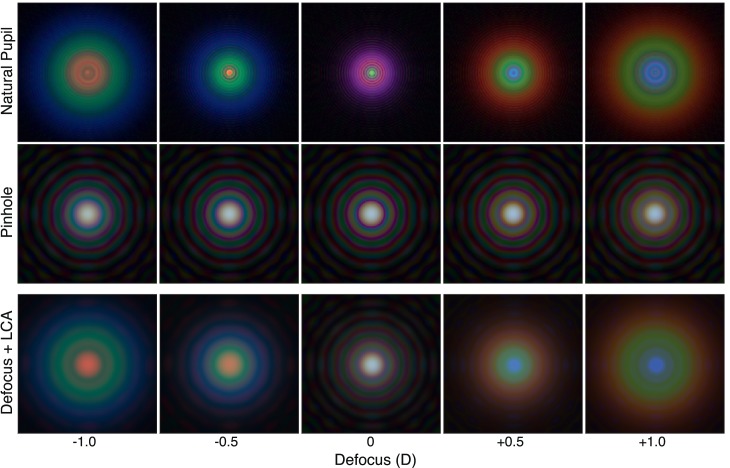
Retinal PSFs for various real and simulated defocus values when viewing through a natural pupil and a pinhole aperture. The eye model incorporated pupil diameter, defocus, diffraction, and LCA. The columns from left to right show PSFs for different defocus values. The upper row shows them for a 4 mm pupil and a real change in focal distance. The middle row shows them for a 1 mm pupil and a real change in focal distance. The bottom row shows them for a 1 mm pupil and a simulated change in focal distance (Defocus + LCA condition) and a 1 mm pupil. Wavelengths simulated for the blue, green, and red primaries are 449, 520, and 617 nm, as in Figure 1.

Despite the superficial similarity, there is a key difference between their simulated conditions (*Luminance Control* and *Chromatic*) and ours (respectively, *Defocus Only* and *Defocus + LCA*). The closed-loop control in their apparatus ensured that accommodative responses had no effect on the retinal image. We did not employ closed-loop control, so responses did affect the retinal image. As a result, the informativeness of various accommodative cues was altogether different in the two experiments. In Kruger's *Chromatic* condition, a response produced no change in the retinal image, so defocus, MFs, HOAs, and astigmatism could not indicate the direction of required response to sharpen the retinal image. LCA was the only informative cue. In our *Defocus + LCA* condition, an accommodative response produced natural changes in the retinal image, so all cues were informative. LCA indicated that the eye should focus in and out in synchrony with the simulated changes; the other cues, including defocus, indicated that the eye should remain accommodated to the actual stimulus distance. Kruger's experiment, therefore, presented no conflict among cues because only one cue—LCA—was informative. Our experiment presented a cue conflict in which LCA indicated that a response was required while the other cues indicated that none was needed. It is somewhat surprising, therefore, that we observed more robust responses to simulated changes than Kruger and colleagues did. We speculate that the difference is our use of natural textures that provide a much broader range of spatial frequencies than their sinewave gratings (Burge & Geisler, 2011).

It is also important to note that Kruger's methodology required changes in rendering *and* changes in focal distance in order to compensate for changes in accommodation. Our technique involved changes in rendering only.

## Removing visual feedback

We next asked how our manipulations of LCA affect accommodation when feedback from accommodative responses is eliminated. We did this by inserting a pinhole aperture in front of the viewer's eye, thereby opening the loop between accommodative response and change in the retinal image. The resulting retinal PSFs are shown in Figure 12. The upper row shows them for real changes in focal distance with a 4 mm pupil. The middle row shows them for real changes in distance with a 1 mm (pinhole) pupil. Notice that the PSFs with a 1 mm aperture do not vary significantly with defocus. The bottom row of Figure 12 shows the retinal PSFs for simulated changes in focal distance (*Defocus + LCA* condition) with a 1 mm pupil. The PSFs now differ across defocus values because the rendered blur varies with simulated focal distance.

This experiment is quite similar to Kruger et al. (1995) in that it makes cues other than LCA uninformative. It is also quite similar to Lee, Stark, Cohen, and Kruger (1999) who used a pinhole to open the accommodative loop and measured responses to simulations of depth-dependent changes in LCA.

### Methods

Six naïve subjects (21–26 years old) participated. Five were female; one was male. All were myopic and wore their prescribed correction during the experiment. All had normal visual acuity when corrected. Without a pinhole, median pupil diameters were 3.24–5.50 mm with an average of 4.32 mm. With a pinhole, they were 3.47–5.95 mm with an average of 5.21 mm (measured of course in the other eye).

We used the same apparatus. Stimuli were viewed with a natural pupil or a 1 mm pinhole placed as close to the cornea as possible. Subjects were instructed to fixate on and accommodate to a textured plane at 1.5 D.

We used step changes in real and simulated focal distance rather than the sinusoidal changes of the previous experiment. There were two image-cue conditions: *Real Change* and *Defocus + LCA*. There were six stimulus changes, ±0.6, ±1.0, and ±1.4 D. A 6 mm pupil was used to model the simulated defocus for the *Defocus + LCA* stimuli. Conditions and stimulus magnitudes were presented in random order. There were also two aperture conditions: *No Pinhole* (viewer's natural pupil) and *Pinhole* (1 mm aperture). *No Pinhole* and *Pinhole* conditions were presented in blocks. In all, 288 trials were presented to each subject: two image-cue conditions, six stimulus magnitudes, two pinhole conditions, and 12 repetitions.

### Results

**Figure 13:**
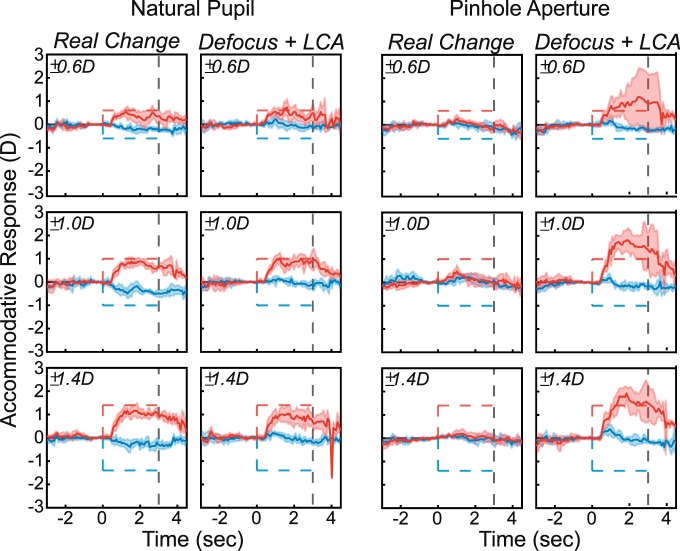
Accommodative responses with natural pupil and pinhole aperture in one subject. Data have been smoothed with a running median calculation with a window of 50 ms. Left: Results with natural pupil. Dashed lines represent the stimulus; red for positive and blue for negative. Shaded regions are median absolute deviations. Thick curves are medians. Left and right columns are for Real Change and Defocus + LCA. Each row shows the data for a different magnitude of change in focal distance: ±0.6, ±1.0, and ±1.4 D. Right: Responses with pinhole aperture in the same subject.

The results for one subject are shown in Figure 13. Results for the others are provided in Supplementary Figure S7. The left half of the figure shows the responses to real and simulated changes in focal distance when viewing through the natural pupil. The subject responded consistently to the changes in actual and simulated focal distance just as in the previous experiment. This subject had an uncorrected myopic refractive error, which meant that she was unable to respond effectively to increases in focal distance. The right half of the figure shows responses when viewing through the pinhole. Here real changes produced no response, as one would expect, because changes in focal distance produced essentially no changes in the retinal image. But simulated changes produced large and variable responses in the direction specified by the LCA cue. The responses to rendered blur with the pinhole were larger than the responses with no pinhole, which is the opposite of what happens with real changes in focal distance.

To assess the statistical reliability of various effects, we subjected the data from 2.5–3 s to a repeated-measures ANOVA with factors of subject, condition, and aperture size, collapsing data across stimulus distance. The response magnitude was divided by the stimulus magnitude to produce an estimate of accommodative gain. We observed statistically significant effects for all factors. Multiple pairwise comparisons were conducted using Tukey contrasts with Bonferroni adjustment. The results showed that accommodation was not significantly different between the *Real Change* and *Defocus + LCA* conditions when no pinhole was present. However, the *Defocus + LCA* condition had significantly greater gains than the *Real Change* condition with a pinhole.

Although the set of cue conflicts we created are nominally the same as those in Kruger et al. (1995) and Lee et al. (1999), the results differ. They observed that responses to simulated changes were smaller than those to real changes. We found that they were actually larger. We again speculate that our stimulus, which contained a broad range of spatial frequencies with spectra similar to natural statistics, provided a better stimulus for accommodation than their sinewave stimulus.

## Color deficiency

The use of LCA requires photoreceptors with different spectral sensitivities, so one expects color-deficient individuals to be less able to employ the cue. Fincham (1953) examined accommodative responses in color-normal and color-deficient observers. The former were trichromats, and the latter were protanopic dichromats (lacking L cones) or deuteranopic dichromats (lacking M cones). He measured accommodation in both groups when the stimulus consisted of two narrow-band primaries (for which LCA is a useful cue) or consisted of just one narrow-band primary (for which LCA is not useful). The great majority of the trichromats accommodated accurately to the two-primary stimuli and less accurately to the one-primary stimuli, which indicates that most color-normal observers use LCA to aid accommodation. The dichromats accommodated less accurately to the two-primary stimuli than the trichromats did. But the dichromats accommodated as accurately to the one-primary stimuli as they did to the two-primary stimuli. Fincham argued that the color-defective observers used other cues to guide accommodation because LCA was mostly unavailable to them. To further examine the use of LCA in driving accommodation, we tested three color-deficient subjects.

### Methods

Subjects were three dichromatic males 19–24 years of age. Two were protanopes, and one was a deuteranope as determined by the HRR Pseudochromatic Color Test. They were tested with the same methods as in the previous experiment.

### Results

**Figure 14:**
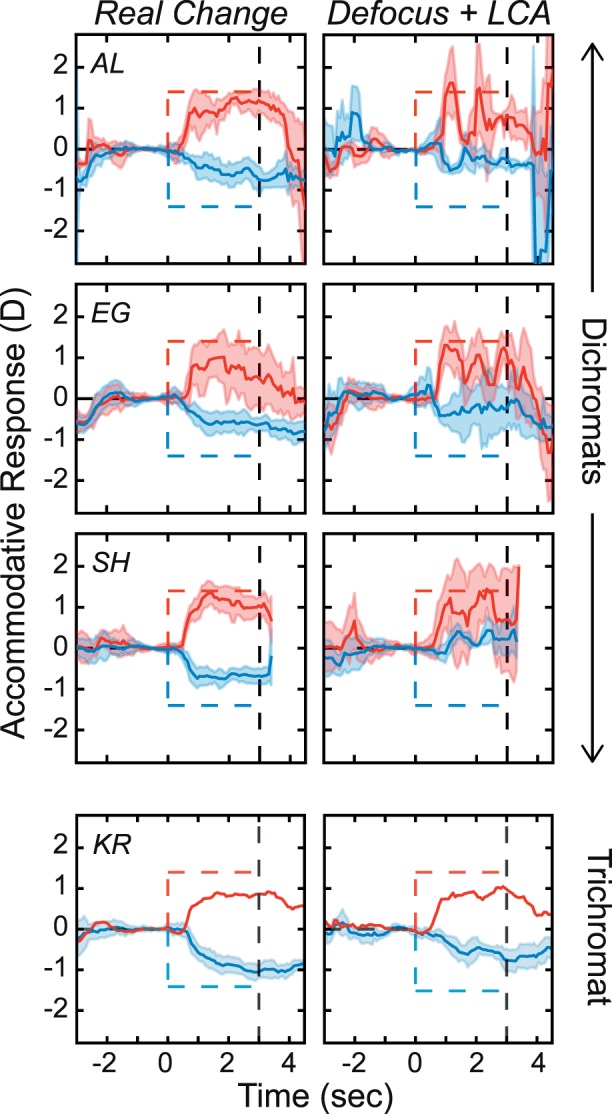
Accommodative responses in three color-deficient, dichromatic subjects and a typical color-normal, trichromatic subject. The first two rows show data from the protanopes and the third row data from the deuteranope. The fourth row shows data from the trichromat. Dashed lines in each panel represent the stimulus. Shaded regions are median absolute deviations; red for positive changes and blue for negative. Thick curves are the medians. Left and right columns are for Real Change and Defocus + LCA. Responses from the deuteranope were inadvertently not recorded after the stimulus went blank; hence, the traces cut off at 3 s.

Figure 14 shows the responses of the color-deficient subjects and a typical color-normal subject in the *Real Change* and *Defocus + LCA* conditions. Protanopes are in the first two rows, the deuteranope in the third row, and the trichromat in the fourth row. Responses of the dichromats in the *Real Change* condition were normal, but responses in the *Defocus + LCA* condition were not: Although dichromats responded in the appropriate direction, they exhibited oscillations and diminished responses that we did not observe in color-normal subjects.

These dichromats are not color-blind because they have two cone types, a characteristic which enables them in principle to use the LCA signal, just less reliably than color normals with three cone types. These results are further evidence that our rendering technique provides a useful signal for accommodation, particularly for the great majority of people who are color-normal.

## Astigmatism

As we mentioned earlier, astigmatism coupled with defocus provides an odd-error signal that could in principle be used to guide accommodation in the correct direction. If an individual had learned the form of astigmatism he/she has, the orientation-dependent blur could in principle indicate the direction the eye needs to accommodate.

Several investigators have shown that subjects with native or induced astigmatism accommodate in a fashion that reduces the effect of the astigmatic error in the retinal image (Byakuno, Okuyama, Tokoro, & Akizawa, 1994; Charman & Whitefoot, 1978; Freeman, 1975). For example, Charman and Whitefoot (1978) measured accommodation when astigmatism was induced optically. The axis of the induced astigmatism was vertical or horizontal and the magnitude varied from 0–6 D. The stimulus was a black-white grating that was vertical, horizontal, or oblique. When the axis of the induced astigmatism was horizontal and the power was +6 D, the focused image of a horizontal contour was 6 D anterior to that of a vertical contour. Thus, the eye had to accommodate much more to focus a vertical as opposed to a horizontal contour. It is important to note that when the grating was vertical or horizontal, all cues (LCA, defocus, HOAs, MFs) signaled the direction and distance to which the eye should accommodate. The authors found that subjects generally accommodated differentially to vertical versus horizontal gratings. In the example, they accommodated ∼5 D more to vertical. The other papers reported similar results. These findings show that the eye does accommodate to reduce blur due to astigmatism, but they do not show that the eye uses astigmatic blur per se to drive accommodation.

We investigated whether such blur can drive accommodation by measuring responses to changes in simulated focal distance instantiated by rendering astigmatic blur where the axis of the blur matched the axis of the subject's native astigmatism. We compared those responses to responses to actual changes in focal distance in the presence of ocular astigmatism. This experiment is conceptually the same as our LCA experiments except that now the only informative cue in the simulated conditions is astigmatic blur.

### Methods

#### Subjects

Three naïve subjects 23–27 years old participated; all were female. We selected these people because their astigmatisms have been uncorrected and because their astigmatic axes are similar in the two eyes. We reasoned that such subjects are more likely to have learned the information astigmatism can provide than subjects with corrected astigmatism or axes that differ in the two eyes (Radhakrishnan, Sawides, Dorronsoro, Peli, & Marcos, 2015). We measured their wavefront aberrations with a Shack-Hartmann sensor (Cheng et al., 2004). Table 1 shows the spherical and cylindrical corrections derived from those measurements. MW and SM do not normally wear optical corrections. MC normally wears a correction for the spherical error, but none for the cylindrical. During the experiment, she wore −5.0 D contact lenses in both eyes to correct her spherical error. The magnitude of astigmatism was large for MW, moderate for MC, and small for SM. We hence highlight results from MW because we expect her to exhibit the clearest effect. Pupil diameters varied from 4.5–5.9 mm across subjects and conditions.

**Table 1:**
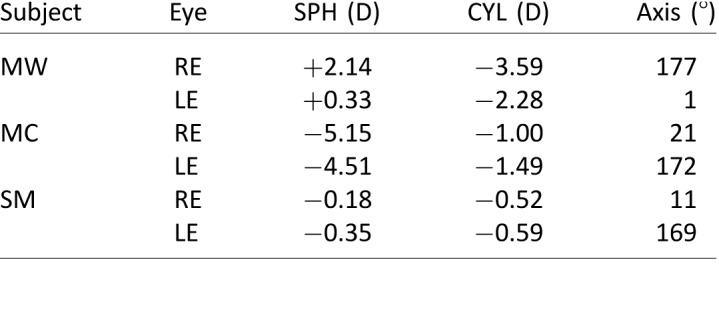
Subjects' wavefront prescriptions at 2.0 D. Notes: SPH = spherical correction; CYL = cylinder correction.

#### Stimuli

The stimuli were again textured fronto-parallel planes. To make LCA uninformative, we illuminated the green primary only (peak = 529 nm, bandwidth = 34 nm). To generate the stimuli for the simulated conditions, we used the same wave-optics method for calculating retinal images as before. We used the wavefront and pupil diameter measurements from each subject in the calculations. The subjects' native astigmatism was optically corrected in the simulated conditions.

#### Apparatus

We used the same apparatus. Stimuli were viewed with a natural pupil or with a 1 mm pinhole placed as close to the cornea as possible. The pinhole opened the loop between accommodation and the retinal image so responses did not change the image on the retina.

#### Procedure

On each trial subjects first fixated and accommodated to the textured plane at 2.0 D for 3 s. Then the experimental stimulus was presented for 3 s at another real or simulated distance. The screen then went blank (uniform green).

There were three image-cue conditions: (a) *Real Change*; the subject's astigmatism was not corrected in this condition, so the native astigmatism of the subjects produced astigmatic blur in the retinal image; (b) *Defocus + Cylinder* in which the simulated focal distance changed in a fashion consistent with the subject's native astigmatism; their astigmatism was corrected optically in this condition so we rendered blur according to the assumption that the subject accommodated at stimulus onset to the midpoint of the interval of Sturm; and (c) *Defocus + Rotated Cylinder* in which the simulated distance changed, but the axis of the simulated astigmatism was rotated by 45° relative to the subject's actual axis; their astigmatism was corrected optically in this condition. Note that the retinal images in this condition were identical to those in the *Defocus + Cylinder* condition except for the 45° rotation, so this condition allowed us to determine whether the alignment of the rendering relative to the native astigmatism mattered. The changes in actual or simulated distance were ±0.6, ±1.0, or ±1.4 D. Conditions and stimulus magnitudes were presented in random order. There were also two aperture conditions: *Natural Pupil* and *Pinhole*. Conditions were presented in blocks. 216 trials were presented to each subject: three image-cue conditions, six stimulus magnitudes, two aperture conditions, and six repetitions.

**Figure 15:**
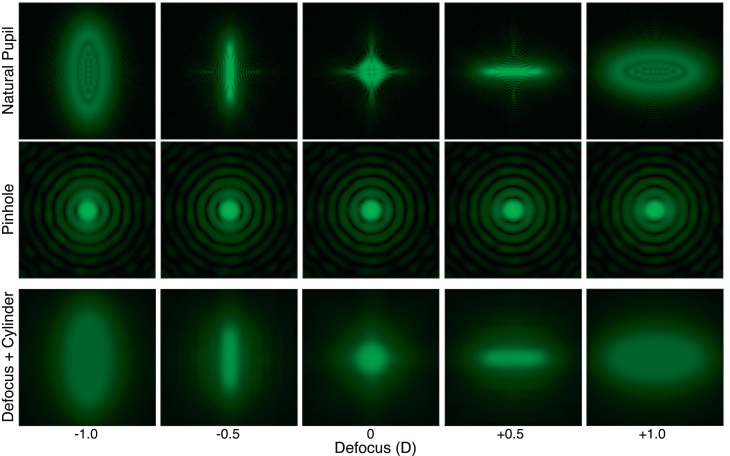
Retinal PSFs for astigmatic eye and various real and simulated defocus values when viewing through a natural pupil and a pinhole aperture. The eye model incorporated pupil diameter, defocus, diffraction, and 0.5 D of astigmatism (axis = 180°). The columns from left to right show PSFs for different defocus values. The upper row shows them for real changes in focal distance with a 4 mm pupil. The middle row shows them for real changes in focal distance with a 1 mm pupil. The bottom row shows them for simulated changes in focal distance (Defocus + Cylinder condition) and a 1 mm pupil. Wavelength is 520 nm.

Figure 15 shows the retinal PSFs calculated from a model eye incorporating defocus, diffraction, and astigmatism. The columns are different real and simulated defocus values. The upper row shows the PSFs for real changes in focal distance with a 4 mm pupil. The middle row shows them for real changes in distance with a 1 mm (pinhole) pupil. Notice that the PSFs change very little in the latter case because the pinhole opens the loop between focal distance and the retinal image. The bottom row shows the retinal PSFs for simulated changes in focal distance (*Defocus + Cylinder* condition) with a 1 mm pupil. Notice that the PSF changes with simulated distance because the rendered blur changes.

### Results

**Figure 16:**
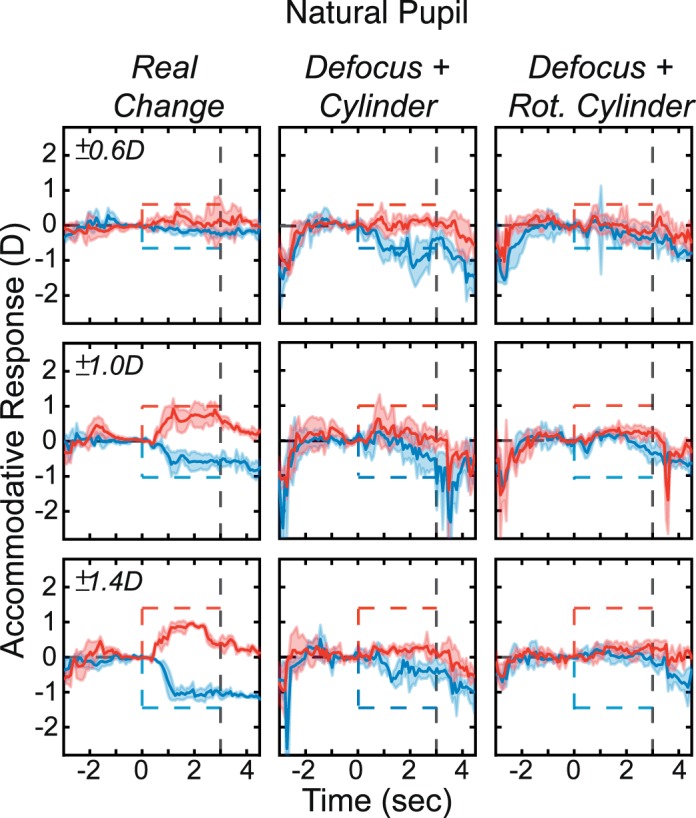
Accommodative responses in subject MW viewing through natural pupil. Responses were subjected to a running median with a window of 50 ms. Dashed lines represent the stimulus. Shaded regions are median absolute deviations. Thick curves are medians. Columns from left to right are for Real Change, Defocus + Cylinder, and Defocus + Rotated Cylinder. Rows from top to bottom are for ±0.6, ±1.0, and ±1.4 D changes.

Figure 16 shows the accommodative responses with natural pupil for MW, the subject with the largest astigmatism. The data from MC, the other subject with a significant astigmatism were similar (Supplementary Figure S8). The data for SM, the subject with insignificant astigmatism, exhibited essentially no response to manipulations of astigmatic blur (Supplementary Figure S8). Responses in subject MW were consistently in the correct direction in the *Real Change* condition. In this case, all cues (except LCA due to the light's narrow spectrum) specified the direction of required response after the step change in focal distance. Responses were usually in the correct direction in the *Defocus + Cylinder* condition, but were noticeably smaller than in *Real Change*. Thus, rendered astigmatic blur drove accommodation, but not very effectively. There were no consistent responses in the *Defocus + Rotated Cylinder* condition, a result which shows that the axis of rendered blur had to be consistent with the subject's native astigmatism to have an effect. We conclude that subjects with 1 D or more of uncorrected astigmatism respond consistently but weakly to astigmatic blur when visual feedback is present.

**Figure 17:**
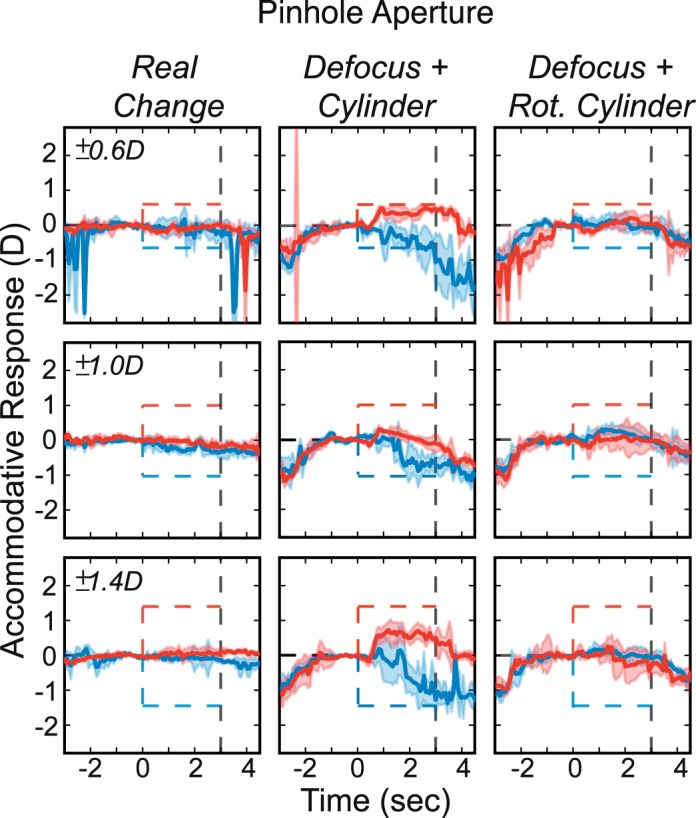
Accommodative responses in subject MW viewing through a pinhole. Same format as Figure 16.

We next examined what happens when visual feedback is removed. Figure 17 shows responses for the same subject when viewing through a pinhole. The data from the other subjects are in Supplementary Figure S8. As expected, little if any response was observed in the *Real Change* condition because the pinhole eliminated changes in the retinal image due to changes in actual focal distance. Reasonably consistent responses were observed in the *Defocus + Cylinder* condition; indeed, they were slightly larger and more consistent than with natural-pupil viewing suggesting that eliminating feedback increases the response to astigmatic blur. The other subject with significant astigmatism (MC) exhibited the same pattern of response (Supplementary Material). The subject with insignificant astigmatism (SM) again exhibited no consistent response to the manipulation of astigmatic blur (Supplementary Material). None of the subjects exhibited consistent responses when the axis of rendered astigmatism was rotated by 45°.

These results show that simulated astigmatic blur can drive accommodation. We believe that this is the first demonstration that astigmatic blur per se can drive accommodation. But unlike our LCA findings, the responses are small and inconsistent.

## Spherical aberration

As we discussed earlier, spherical aberration can in principle provide directional information to guide accommodation. We examined whether it is actually used by creating stimuli with spherical-aberration effects consistent with stimuli either nearer or farther than the eye's current focus distance. Most eyes have positive spherical aberration when accommodated far and negative spherical aberration when accommodated near (Cheng et al., 2004; Tarrant et al., 2010). We took such changes into account by tailoring the rendering to each subject's native aberration at the focus distance of the initial stimulus.

### Methods

#### Subjects

Three naïve subjects 24–28 years old participated; all were female. We measured their wavefront aberrations with a Shack-Hartmann sensor with a stimulus distance of 3.0 D. The prescriptions from the wavefront measurements are provided in Table 2. None of the subjects had astigmatisms greater than 0.5 D in their left eye when wearing their habitual correction. AG does not wear correction. BB wears contact lenses to correct myopia and astigmatism (prescription RE: −8.50/−1.75 × 180, LE: −7.50/−2.25 × 180). PS wears contact lenses to correct myopia (prescription RE: −6.25/0, LE: −5.75/0). Pupil diameters varied from 4.8–6.2 mm. Subjects had normal visual acuity (BB and PS when corrected).

**Table 2:**
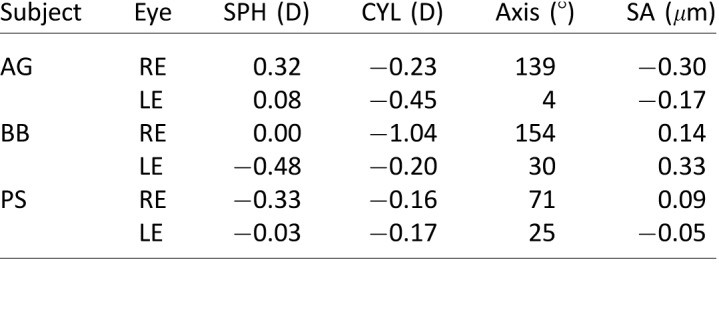
Subjects' wavefront prescriptions at 3.0 D. Notes: SPH = spherical correction; CYL = cylinder correction; SA = spherical aberration.

#### Stimuli

Stimuli were again textured planes. To make LCA uninformative, we illuminated the green primary only. To generate the stimuli for the simulated conditions, we used the same wave-optics method for calculating retinal images as before. In this experiment, however, we only manipulated spherical aberration and defocus using the wavefront and pupil-diameter measurements from each subject in the calculations.

#### Apparatus

We used the same apparatus. Stimuli were viewed with a 1 mm pinhole placed as close to the cornea as possible. Subjects' native aberrations (including spherical aberration) were not optically corrected, but the pinhole viewing eliminated their contribution to the retinal image and opened the accommodative loop so responses had no effect on the retinal image.

#### Procedure

There were three image-cue conditions: (a) *Real Change*; (b) *Defocus Only* in which focal distance did not change, but the rendered blur changed by the amount commensurate with the magnitude of the change in simulated distance (equivalent to conventional rendering); and (c) *Defocus + Spherical Aberration* in which the focal distance did not change, but the simulated distance changed in a fashion consistent with the subject's native spherical aberration and the defocus. The changes in actual and simulated distances were ±0.6, ±1.0, or ±1.4 D. Conditions and stimulus magnitudes were presented in random order. 180 trials were presented to each subject: three image-cue conditions, six stimulus magnitudes, and 10 repetitions.

The subject first fixated and accommodated to the plane at 3.0 D for 3 s. The texture was sharp in the *Real Change* and *Defocus Only* conditions. In the *Defocus + Spherical Aberration* condition, the initial texture was rendered according to the individual subject's spherical aberration when accommodated to 3.0 D. After the presentation of the plane at that distance, the experimental stimulus was presented for 3 s at another real or simulated distance. The screen then went blank (uniform green).

### Results

**Figure 18:**
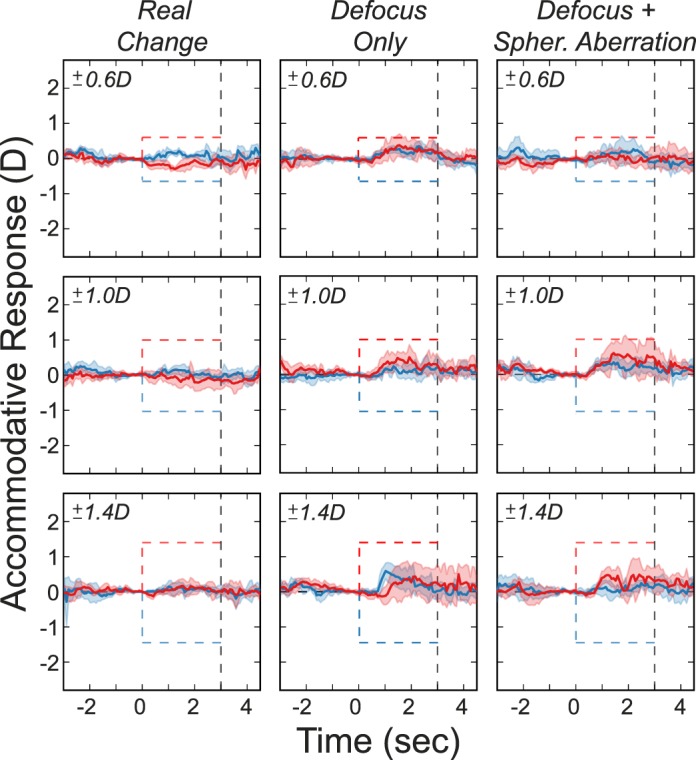
Accommodative responses in subject PS viewing through pinhole aperture. Responses were subjected to a running median with a window of 50 ms. Dashed lines represent the stimulus. Shaded regions are median absolute deviations. Thick curves are the medians. Columns from left to right are for Real Change, Defocus Only, and Defocus + Spherical Aberration. Rows from top to bottom are for ±0.6, ±1.0, and ±1.4 D changes.

The results from one subject are shown in Figure 18. The results from the other two were similar and are provided in Supplementary Figure S9. There were no consistent responses in any of the conditions. We expected no response in the *Real Change* condition because the pinhole opened the loop between accommodation and the retinal image. We also expected no response in the *Defocus Only* condition because the defocus inserted in the stimulus carries no information about the direction of required response. The critical condition was the *Defocus + Spherical Aberration* condition where the direction of required response was in principle specified in the retinal image. The fact that none of the subjects exhibited consistent responses in this condition suggests that defocus plus spherical aberration is unable to elicit accommodative responses. We conclude that subjects with measurable spherical aberration do not respond consistently to blur produced by defocus plus spherical aberration when no visual feedback is present.

## Discussion

In this paper we described rendering for “perceptual realism” rather than “photorealism.” Specifically, we examined how depth-dependent optical aberrations in the human eye provide information that could potentially help guide accommodation. We pointed out that these depth-dependent effects are not reproduced in the rendering techniques that are commonly used in computer graphics and vision science. To address this, we developed a rendering method that reproduces the effects reasonably accurately. Using this method, we showed that color-correct blur rendering (taking the eye's chromatic aberration into account) is quite effective in stimulating accommodation. We found that correctly rendering astigmatism was less effective and that correctly rendering spherical aberration had no effect. In the following sections, we investigate why the accommodative system responds robustly to color-correct rendering but not to the rendering of the other aberrations. We discuss the usefulness of LCA in spectrally varying environments. And we discuss potential applications.

### Predicting responses to different rendering techniques

We observed robust responses to simulated distance in the LCA experiments (Figure 10), weaker but consistent responses in the astigmatism experiment (Figures 16 and 17), and no responses in the spherical aberration experiment (Figure 18). These results can be explained by the relative strengths of the signals being manipulated. From the wavefront measurements in each subject, we calculated signed differences between PSFs with the simulated aberrations for positive and negative defocus (Table 3):

\newcommand{\bialpha}{\boldsymbol{\alpha}}\newcommand{\bibeta}{\boldsymbol{\beta}}\newcommand{\bigamma}{\boldsymbol{\gamma}}\newcommand{\bidelta}{\boldsymbol{\delta}}\newcommand{\bivarepsilon}{\boldsymbol{\varepsilon}}\newcommand{\bizeta}{\boldsymbol{\zeta}}\newcommand{\bieta}{\boldsymbol{\eta}}\newcommand{\bitheta}{\boldsymbol{\theta}}\newcommand{\biiota}{\boldsymbol{\iota}}\newcommand{\bikappa}{\boldsymbol{\kappa}}\newcommand{\bilambda}{\boldsymbol{\lambda}}\newcommand{\bimu}{\boldsymbol{\mu}}\newcommand{\binu}{\boldsymbol{\nu}}\newcommand{\bixi}{\boldsymbol{\xi}}\newcommand{\biomicron}{\boldsymbol{\micron}}\newcommand{\bipi}{\boldsymbol{\pi}}\newcommand{\birho}{\boldsymbol{\rho}}\newcommand{\bisigma}{\boldsymbol{\sigma}}\newcommand{\bitau}{\boldsymbol{\tau}}\newcommand{\biupsilon}{\boldsymbol{\upsilon}}\newcommand{\biphi}{\boldsymbol{\phi}}\newcommand{\bichi}{\boldsymbol{\chi}}\newcommand{\bipsi}{\boldsymbol{\psi}}\newcommand{\biomega}{\boldsymbol{\omega}}\begin{equation}\tag{9}\int\limits_{_{psf}} {\left| {PS{F_{pos}}(x,y) - PS{F_{neg}}(x,y)} \right|} \,{\rm{d}}x\,{\rm{d}}y\end{equation}

where *PSF_pos_* is the point-spread function for an eye with the listed aberration when defocus is positive and has a particular magnitude, and *PSF_neg_* is for negative defocus of the same magnitude. We calculated the signal strengths for LCA, astigmatism, and spherical aberration assuming a 5 mm pupil, which is close to the measured value in the experiments. For astigmatism and spherical aberration, we assumed 520 nm, consistent with the experiments. For LCA, we assumed 449, 520, and 617 nm, consistent with those experiments. The volumes of the PSFs were first normalized to 1. We then computed the differences between *PSF_pos_* and *PSF_neg_* and integrated to generate one value for each comparison. The results are provided in Table 3. For LCA, we added the values for red and blue because the visual system has access to both signals. As you can see, the largest difference is observed for LCA (red + blue), followed by astigmatism, and then spherical aberration. These values agree well with our experimental results which showed robust responses to LCA, modest but consistent responses to astigmatism, and no response to spherical aberration (Figures 16 and 17 and Supplementary Figure S8; Figure 18 and Supplementary Figure S9). We conclude that the effectiveness of LCA, astigmatism, and spherical aberration in driving accommodation depends on the strength of those signals.

**Table 3:**
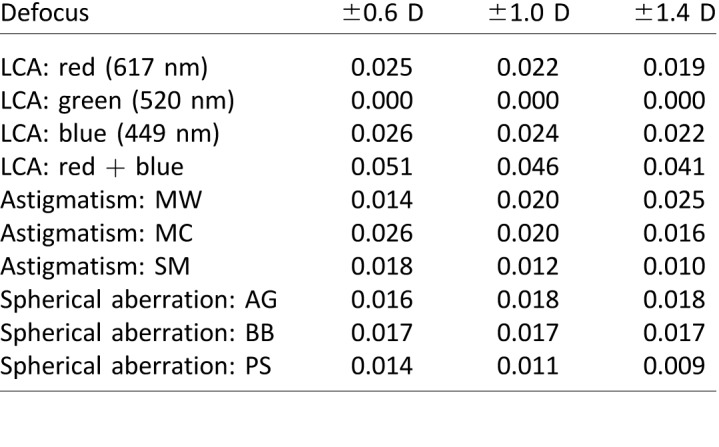
Differences in aberrated PSFs for positive and negative defocus (Equation 9).

### Accommodation to LCA

We observed very robust responses to changes in simulated focal distance when the rendering incorporated LCA. Why did this occur?

The right panel of Figure 6 shows the sizes of the blur kernels to use in the displayed image in order to generate retinal images similar to those produced by viewing the real world. There are different sizes for each color primary because of the viewer's LCA. When we simulate that the object distance is the same as the eye's current focus distance, red, green, and blue are displayed sharp so the LCA of the viewer's in-focus eye produces a relatively sharp retinal image in green and blurred images in red and blue (Figure 6). When we simulate that the object is nearer than current focus (positive defocus), blue is displayed sharper than green which is displayed sharper than red (Figure 6). Our results and those of Cholewiak et al. (2017) show that presenting such a stimulus causes the viewer's eye to accommodate nearer such that it becomes focused *in front of* the screen. The viewer's native LCA then causes blue to be blurred more than red and this partially compensates for the difference in the displayed image. Said another way, the accommodative response restores the usual balance between the blurs observed at short and long wavelengths when the eye is in focus. When we instead simulate that the object is farther than current focus, red is displayed sharper than green which is displayed sharper than blue (Figure 6). Our results and those of Cholewiak et al. (2017) show that this stimulus causes the viewer to accommodate farther such that focus is now *behind* the screen. The viewer's LCA then causes red to be more blurred than blue, which again partially compensates for the difference in the displayed image.

**Figure 19:**
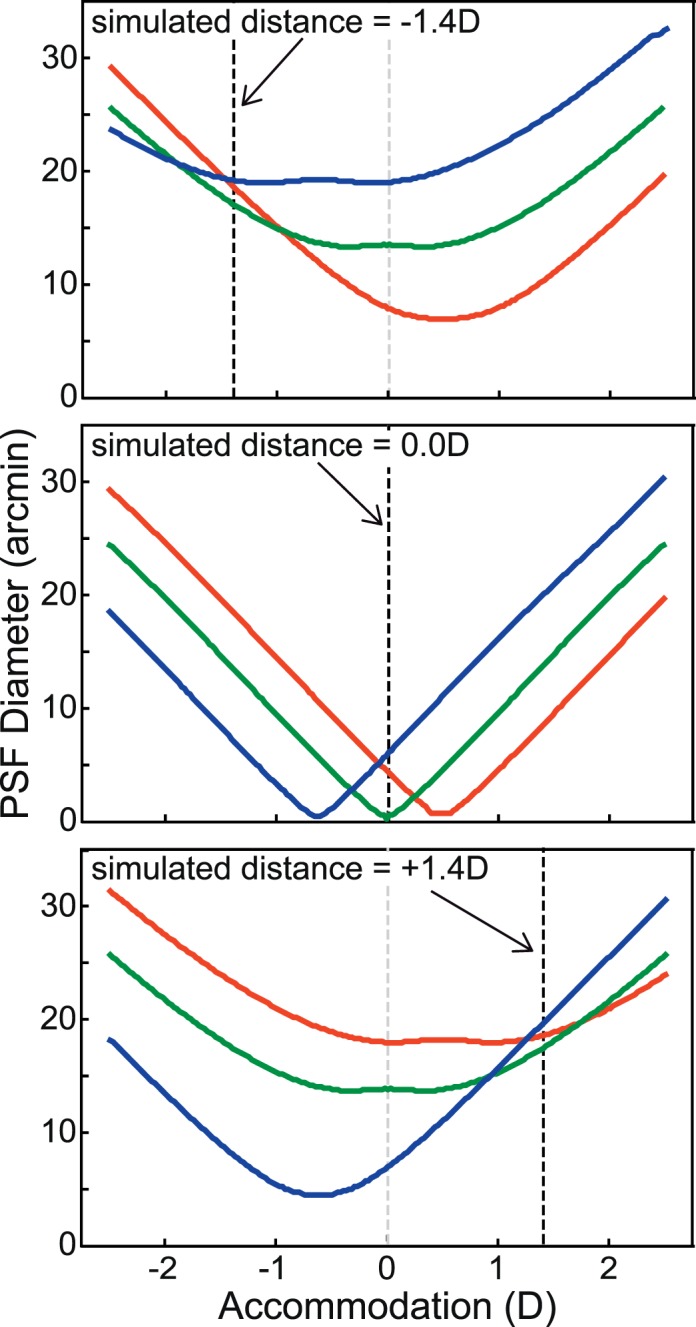
Retinal PSF diameters for the R, G, and B primaries generated by different combinations of simulated focal distance and accommodative response. Top, middle, and bottom panels show those diameters for simulated distances of –1.4, 0, and +1.4 D, respectively. Each panel plots the diameter of the PSF at the retina as a function of accommodation. The diameters were calculated using encircled energy. We first calculated the energy of the whole PSF. We then fit circles of increasing diameter, centered on the PSF centroid, and measured the energy within each circle until we found the diameter containing 50% of the total energy. The red, green, and blue curves are those diameters for the R, G, and B primaries of our display. The dashed black lines represent the simulated focal distances. The dashed gray lines represent the nominal accommodative distance at stimulus onset.

These accommodation-dependent effects on the retinal image are illustrated in Figure 19. Each panel plots diameters of retinal PSFs as a function of accommodative distance. The upper, middle, and bottom panels show those diameters for simulated distances of –1.4, 0, and +1.4 D (each indicated by a dashed black line). In each case, accommodating to the simulated distance creates roughly equivalent blurs at short and long wavelengths. We hypothesize that this is the system's strategy: Adjust accommodation such that middle wavelengths are less blurred than short and long wavelengths, and such that short and long wavelengths are blurred by similar amounts. This strategy could be instantiated by comparing responses among color-opponent neural mechanisms (Flitcroft, 1990; Shapley & Hawken, 2011). Ironically, the strategy of balancing blurs at short and long wavelength comes at the cost of overall image quality: From Figure 19, one can see that the retinal image would be sharper if the eye accommodated to the distance of the screen. With a pinhole, accommodation does not affect the retinal image, so the eye is unable to achieve this balance between blurs at short and long wavelengths and hence accommodates by larger amounts than with a natural pupil (Figure 13).

**Figure 20:**
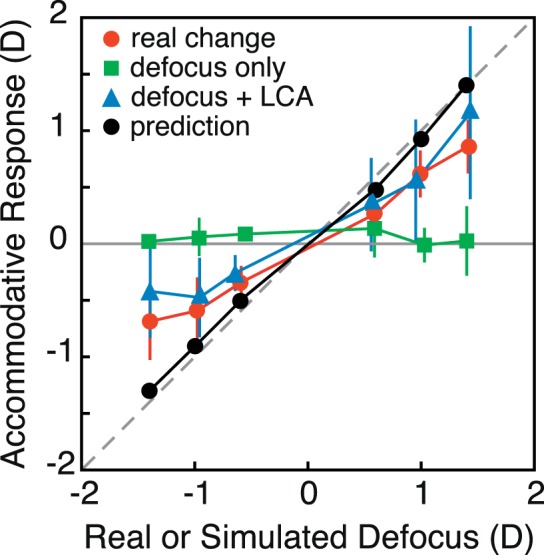
Observed and predicted accommodative responses. Responses to step changes of ±0.6, ±1, and ±1.4 D are plotted as a function of real or simulated change in focal distance. Red circles, green squares, and blue triangles represent the data for the Real Change, Defocus Only, and Defocus + LCA conditions. The plotted values were obtained by calculating the median response for each condition and subject for the final second of stimulus presentation (Cholewiak et al., 2017). Then the medians were averaged across subjects. Black circles represent the predicted responses based on the model depicted in Figure 19. The predicted response is the value that produced the same difference in PSF diameters for R and B as occurred when the simulated focal distance and response were 0 D.

Figure 20 compares predicted responses for all simulated distances to observed responses at the same distances. The predictions were derived by finding the accommodative response that yielded the same difference in PSF diameters for R and B that occurred when the simulated distance and accommodative distance are 0 D (middle panel, Figure 19). The observed and predicted responses are similar, which shows that this model provides a reasonable account of how color-correct rendering evokes robust accommodative responses.

Accommodation is conventionally considered a control system designed to maximize image sharpness, where sharpness is quantified by various image-quality metrics including RMS wavefront error, PSF width, and visual Strehl ratio (Cheng, Bradley, & Thibos, 2004). Our results are inconsistent with this view. Instead of maximizing image sharpness as defined by those metrics, accommodative responses to simulated changes in focal distance (specifically, *Defocus + LCA*) actually reduce sharpness. Instead they create roughly equivalent blurs at short and long wavelengths. In the natural environment, such a strategy will generally achieve high image quality. By decoupling cues, we showed that LCA is a powerful determinant of human accommodation, so powerful that the system will tolerate a decrease in perceived sharpness in order to maintain roughly equivalent blurs at short and long wavelengths.

### Usefulness of LCA in natural scenes

Our stimuli were grayscale images that were split into R, G, and B components for implementing color-correct rendering. It is reasonable to ask if the rendering method would drive accommodation as effectively if the images were more saturated. We did not examine this question directly, but computational work on estimating defocus from natural images has shown that LCA is a critical cue for accurate performance when modeling the human eye with its significant LCA (Burge & Geisler, 2011) or when modeling a camera with minimal LCA (Burge, 2017). Thus, we are confident that color-correct rendering would be effective for most natural images. It would not be effective for image patches with only one saturated color.

### Pupil diameter

In our experiments we tried to make the pupil diameter assumed in rendering match the actual diameter. There is reason to believe, however, that one can still drive accommodation effectively if the assumed pupil diameter does not closely approximate the actual diameter.

When LCA is doubled or halved in magnitude, accommodative responses are largely unaffected (Kruger et al., 1993). This finding makes sense. From Equation 1, the diameter of the blurred image of a point object is

$$\newcommand{\bialpha}{\boldsymbol{\alpha}}\newcommand{\bibeta}{\boldsymbol{\beta}}\newcommand{\bigamma}{\boldsymbol{\gamma}}\newcommand{\bidelta}{\boldsymbol{\delta}}\newcommand{\bivarepsilon}{\boldsymbol{\varepsilon}}\newcommand{\bizeta}{\boldsymbol{\zeta}}\newcommand{\bieta}{\boldsymbol{\eta}}\newcommand{\bitheta}{\boldsymbol{\theta}}\newcommand{\biiota}{\boldsymbol{\iota}}\newcommand{\bikappa}{\boldsymbol{\kappa}}\newcommand{\bilambda}{\boldsymbol{\lambda}}\newcommand{\bimu}{\boldsymbol{\mu}}\newcommand{\binu}{\boldsymbol{\nu}}\newcommand{\bixi}{\boldsymbol{\xi}}\newcommand{\biomicron}{\boldsymbol{\micron}}\newcommand{\bipi}{\boldsymbol{\pi}}\newcommand{\birho}{\boldsymbol{\rho}}\newcommand{\bisigma}{\boldsymbol{\sigma}}\newcommand{\bitau}{\boldsymbol{\tau}}\newcommand{\biupsilon}{\boldsymbol{\upsilon}}\newcommand{\biphi}{\boldsymbol{\phi}}\newcommand{\bichi}{\boldsymbol{\chi}}\newcommand{\bipsi}{\boldsymbol{\psi}}\newcommand{\biomega}{\boldsymbol{\omega}}\beta = A\left| {{1 \over {z_0}} - {1 \over {z_1}}} \right| = A\left| {\Delta D} \right|$$

The focal difference between two wavelengths (Figure 2) can be expressed in diopters. From this equation, one can see that the same chromatic effect at the retina can occur with various combinations of change in focal distance and pupil diameter: That is, halving or doubling the magnitude of LCA is equivalent to halving or doubling pupil diameter. An optimal defocus estimator that uses LCA behaves in the same fashion: It estimates half the depth variation (in diopters) when it is presented images captured with twice the aperture size that was used in the training set (Burge & Geisler, 2011).

Without knowing pupil diameter, the visual system could not use LCA to determine the magnitude of the response required to refocus the image even though LCA could still be used to determine the required direction.

### Practical uses for correct blur rendering

Important perceptual and ergonomic issues arise with stereoscopic displays, such as the head-mounted displays used for virtual reality (VR) and augmented reality (AR). Many of the issues are due to the vergence-accommodation conflict. Vergence and accommodation are neurally coupled (Schor, 1992), which is beneficial in the real world where the distances to which the eyes should converge and accommodate are always the same. But the coupling is broken by conventional stereoscopic displays because such displays require the viewer to converge to one distance (that of the virtual object) while accommodating to another (the display screen). The resulting conflict causes visual discomfort (Hoffman, Girshick, Akeley, & Banks, 2008; Koulieris, Bui, Banks, & Drettakis, 2017; Lambooij, Fortuin, Heynderickx, & IJsselsteijn, 2009; Shibata, Kim, Hoffman, & Banks, 2011), reductions in performance (Akeley, Watt, Girshick, & Banks, 2004; Johnson et al., 2016; Konrad, Cooper, & Wetzstein, 2016; Maiello, Chessa, Solari, & Bex, 2014), and distortions of perceived depth (Watt, Akeley, Ernst, & Banks, 2005). By better understanding how to stimulate accommodation, the work presented here provides an opportunity to drive accommodation and thereby minimize the vergence-accommodation conflict.

One can reproduce the natural relationships between blur, accommodation, disparity, and vergence by coupling focus-adjustable lenses placed between the eyes and display screen with the virtual content (Johnson et al., 2016; Konrad et al., 2016). If the screen and virtual stereoscopic content are at distance *z*_0_, one sets the adjustable lens to zero power (i.e., infinite focal distance), and the eyes naturally accommodate and converge to that distance creating a single, sharp retinal image. If we want to present content at a greater distance, binocular disparity is changed thereby stimulating the eyes to diverge and the power of the adjustable lens is increased, causing the eyes to accommodate farther than before to focus on the screen. This minimizes conflict between vergence and accommodative responses, thereby improving ergonomic and perceptual performance (Johnson et al., 2016; Konrad et al., 2016; Koulieris et al., 2017).

By using color-correct blur rendering one can drive accommodation to the next fixated distance while the graphics system updates rendering appropriately for that next distance. Thus, adding our rendering technique to displays with focus-adjustable lenses offers a great opportunity for driving accommodation more quickly and for creating more realistic imagery. But our technique requires deconvolution or some similar process and this makes real-time response difficult. One can greatly speed up calculations by using GPU computing, OpenGL shader-based approximations, and other methods. Such approximations produce incorrect results where the depth gradient is large (e.g., occlusions, reflections). We are currently developing methods that enable real-time updating and are measuring how effectively they can be used to drive accommodation and create realistic depth appearance (Cholewiak et al., 2018).

One could also simplify the blur-rendering computation by nulling the viewer's LCA optically. Then the rendering becomes a straight-forward calculation in which different focal planes are simulated for each color primary. One could null LCA by using a focus-adjustable lens that is synchronized to sequential presentation of the B, G, and R primaries. One could also null LCA with a static lens designed to reverse the eye's native LCA (Kruger et al., 1993).

## Supplementary Material

Supplement 1
