# Supplementary material for: Creating correct blur and its effect on accommodation
Source: J Vis. 2018 Sep 4;18(9):1. doi: 10.1167/18.9.1 (PMC6126933; doi:10.1167/18.9.1)
Supplement: Supplement 1 [file jovi-18-08-07_s01.pdf]

Supplementary Material

Apparatus

Color primaries

The stimuli were projected onto a screen by a DLP projector (Texas Instruments LightCrafter 4710 Evaluation Module). The LED light sources were produced by OSRAM, with the following specifications provided by the manufacturer; Red: LE A P1W; Green: LE CG P1A; Blue: LE B P1W. We placed a triple-bandpass filter (Chroma 69002m) between the projector and the screen to create narrower spectra. We measured the resulting emission spectra of the three primaries through the autorefractor's hot mirror using a Photo Research PR-650 SpectraScan Colorimeter. These spectra are shown in Fig. S1. Table S1 shows the peak wavelength, bandwidth (full width at half height), luminance, and radiance (at highest intensity for each primary).

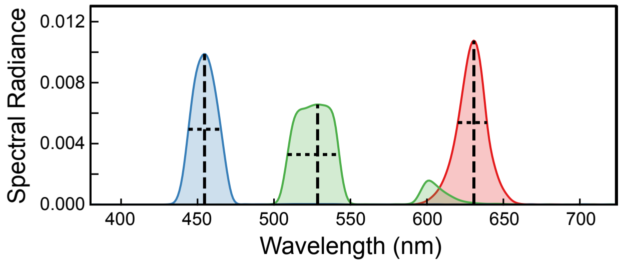

Figure S1: Spectral radiance as a function of wavelength measured from the subject's viewpoint, described in Table S1. Dashed vertical lines are peak radiances for each primary and dotted horizontal lines are bandwidths (50% of peak radiance).

Table S1: Measured Experimental Color Spectra

Primary Color	Peak (nm)	Bandwidth (nm)	Luminance (cd / m^2)	Radiance ($W \cdot sr^{-1}$ m^{-2} nm^{-1})
Red	631	19.1	46.1	0.240
Green	529	33.9	128.1	0.253
Blue	455	21.3	8.2	0.215

Defocus Kernel Diameters Optimized for Individuals

The calculated blur kernel diameters in Fig. 6 are based on an eye model with defocus and LCA only. In Fig. S2, we examined how other aberrations affect the calculated kernels. The displayed images were produced using the calculated kernel (Fig. 3) with defocus, LCA, and the first 21 Zernike modes, excluding piston, tip, and tilt, for our subjects' eyes. The aberrations were measured using a Shack-Hartmann wavefront sensor. The wavelengths for the R, G, and B primaries are 617, 520, and 449nm; pupil diameter is 4mm.

Example Stimuli

Fig. S3 provides cropped versions of example stimuli.

LCA Sinusoidal Stimulus

Figs. S4, S5, and S6 provide the data from the nine subjects whose data are not shown in Fig. 10.

LCA Step Stimulus with Pinhole

Fig. S7 provides the data from the five subjects whose data are not shown in Fig. 13.

Astigmatism

Fig. S8 provides the data from the two subjects whose data are not shown in Figs. 16 and 17.

Spherical Aberration

Fig. S9 provides the data from the two subjects whose data are not shown in Fig. 18.

Retinal PSF Diameters with Aberrated Eyes

Fig. S10 shows the effect of incorporating other aberrations (first 21 Zernike modes, excluding piston, tip, and tilt) into the eye model. The format is the same as the encircled energy plots in Fig. 19. Retinal PSF diameters for displayed images calculated for defocus and LCA only (e.g., Fig. 8) are indeed altered when the other aberrations are included. But the effect is quite small for most observers because the diameter of the encircled energy is dominated by defocus.

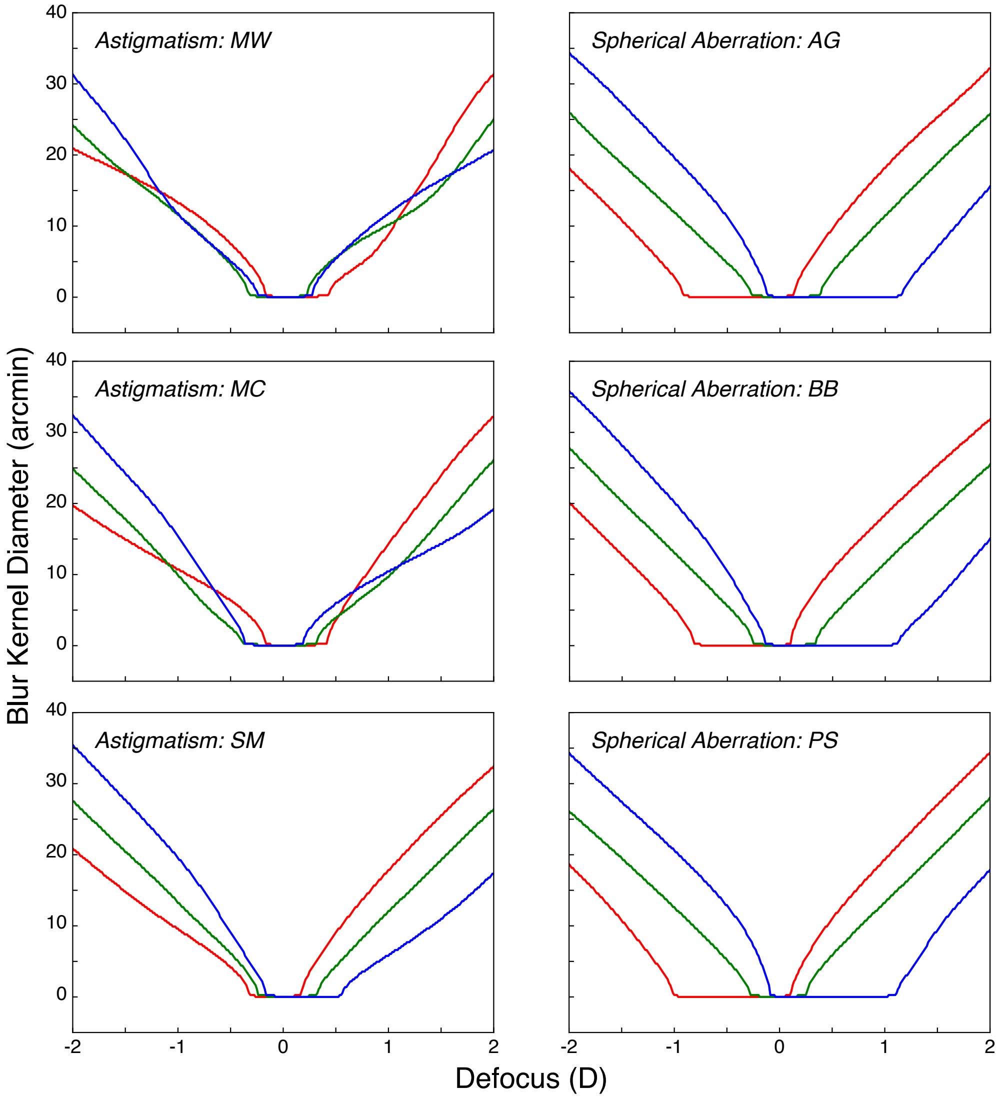

Figure S2: Defocus and blur kernels for rendering when all aberrations are included. The format is the same as the right panel of Fig. 6. The panels show the diameters of the calculated blur kernels as a function of simulated defocus. There is a small effect of adding the other aberrations in two of the astigmatic subjects (MW & MC) for small defocus values where the uncorrected astigmatism has a noticeable effect. At larger defocus values, the dominant signal is defocus so the curves become very similar to those in Fig. 6.

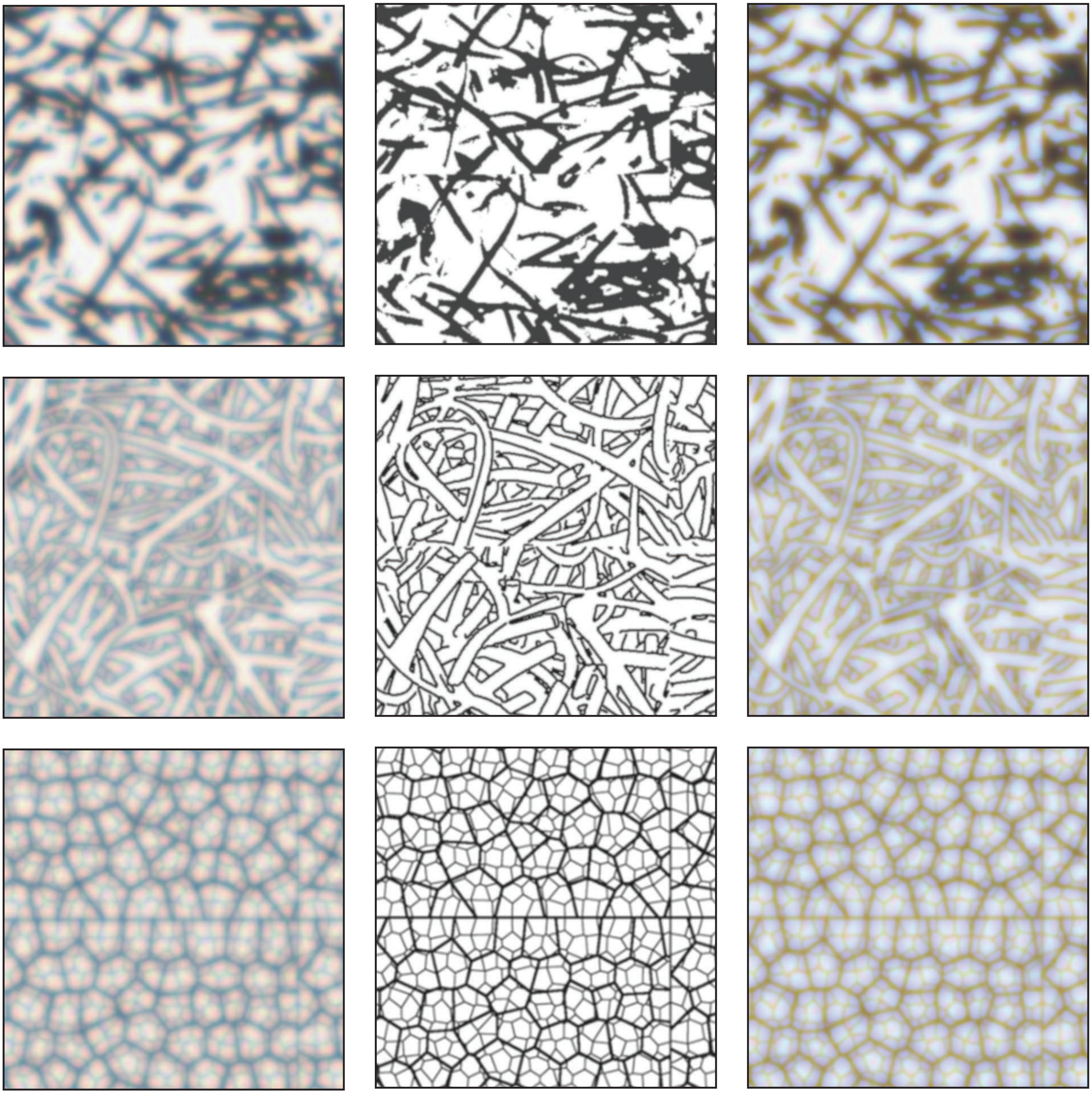

Figure S3: Cropped versions of stimuli generated by our color-appropriate rendering method. Images in the left column are simulated to be farther than the current focus distance of the eye. Images in the center column are simulated to be at the current eye focus distance. Images in the right column are simulated to be nearer than current eye focus. These should be viewed from a distance of 5.6 times the height of the individual panels.

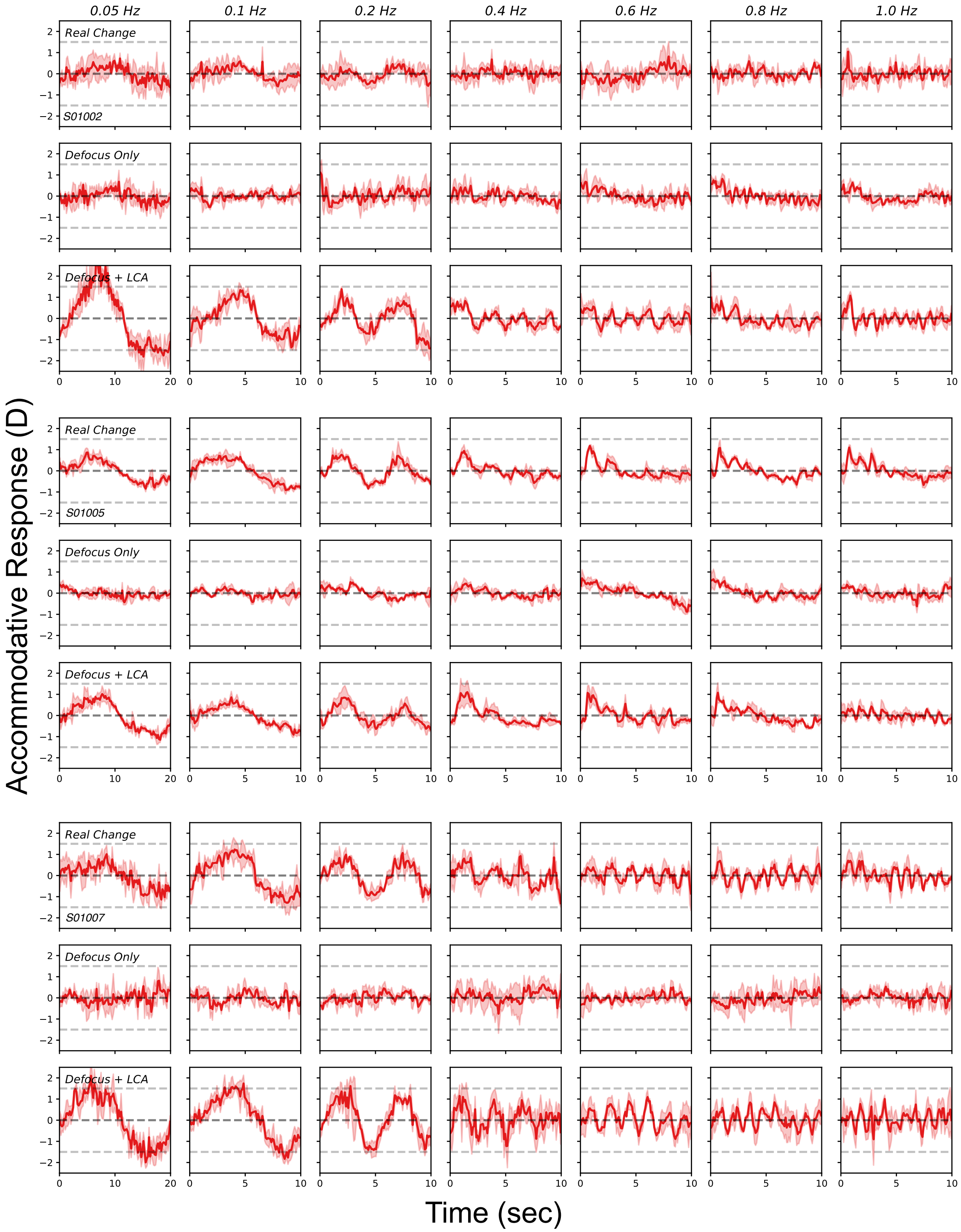

Figure S4: Data from three additional subjects in the LCA experiment with sinusoidal stimuli. The format of each set of data is the same as Fig. 10.

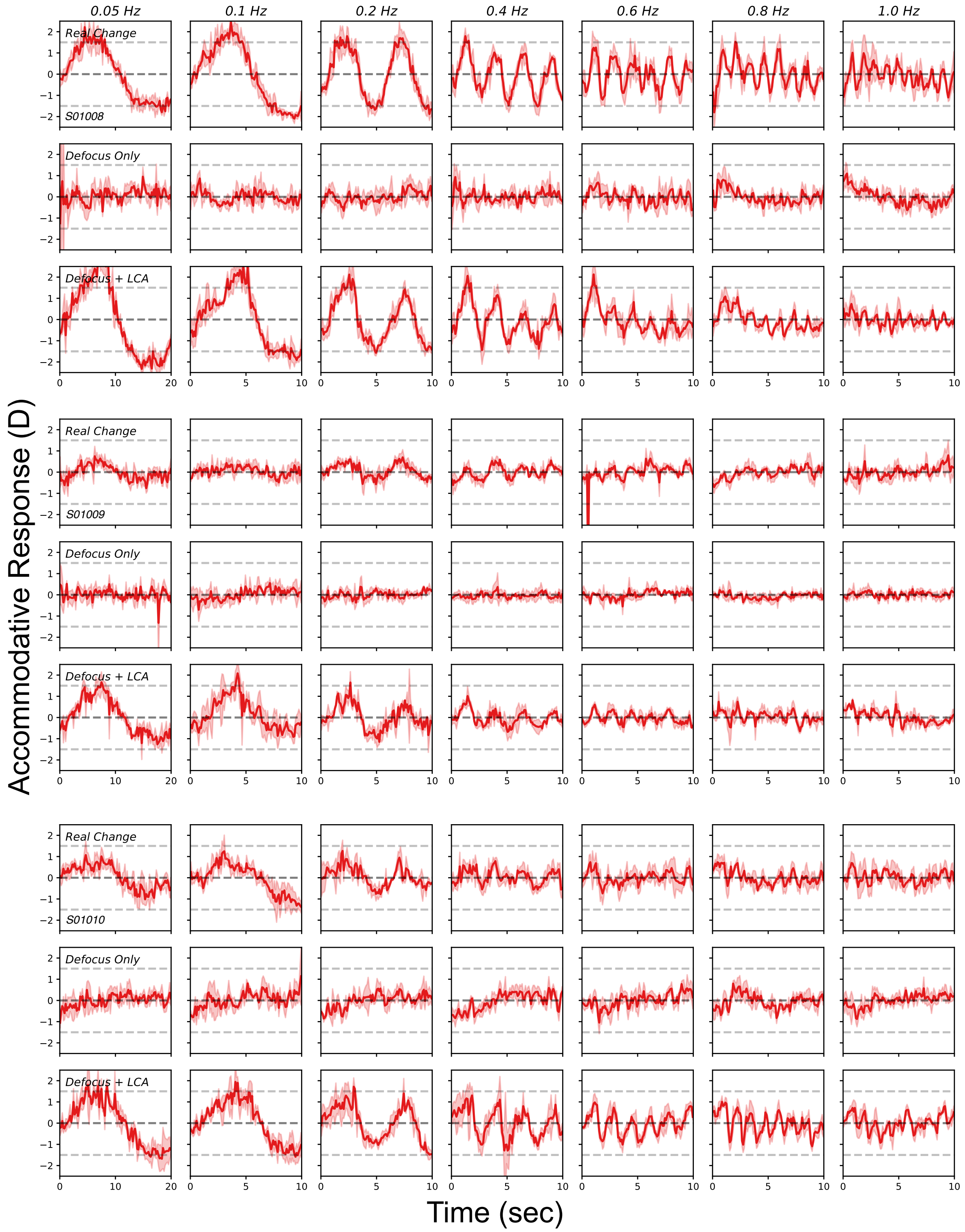

Figure S5: Data from another three subjects in the LCA experiment with sinusoidal stimuli.

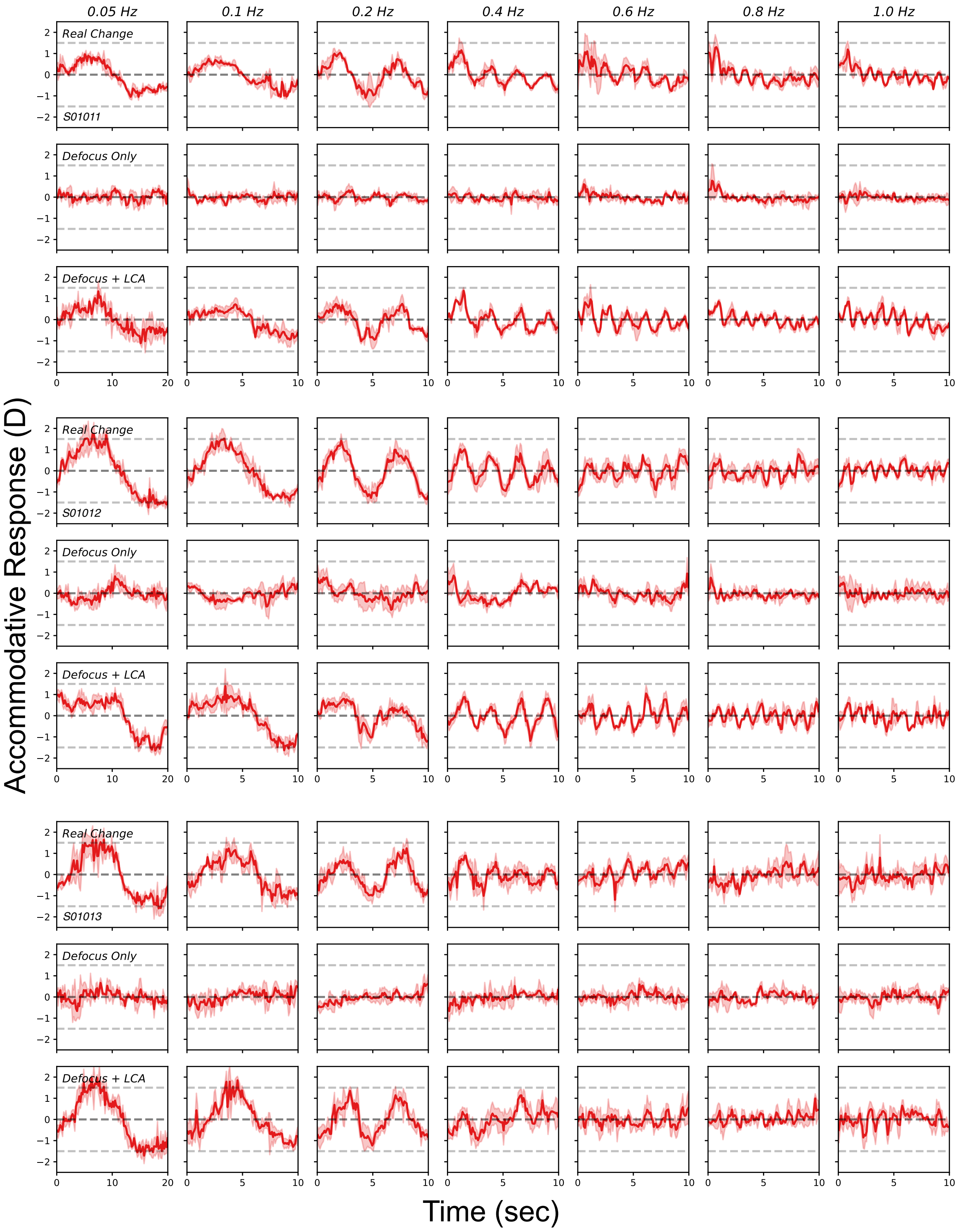

Figure S6: Data from another three subjects in the LCA experiment with sinusoidal stimuli.

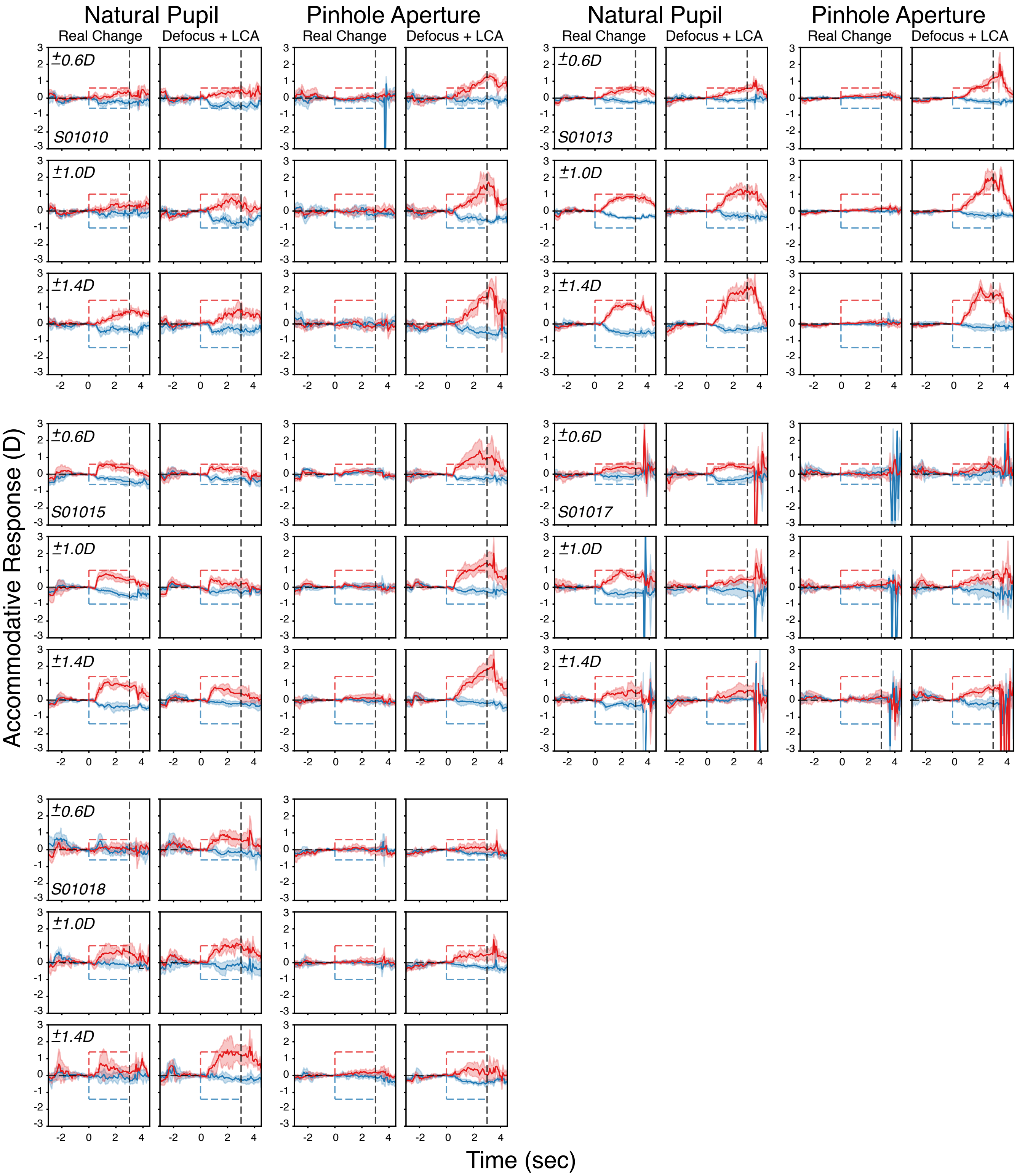

Figure S7: Data from the other five subjects in the LCA experiment with and without a pinhole aperture. The format is the same as Fig. 13.

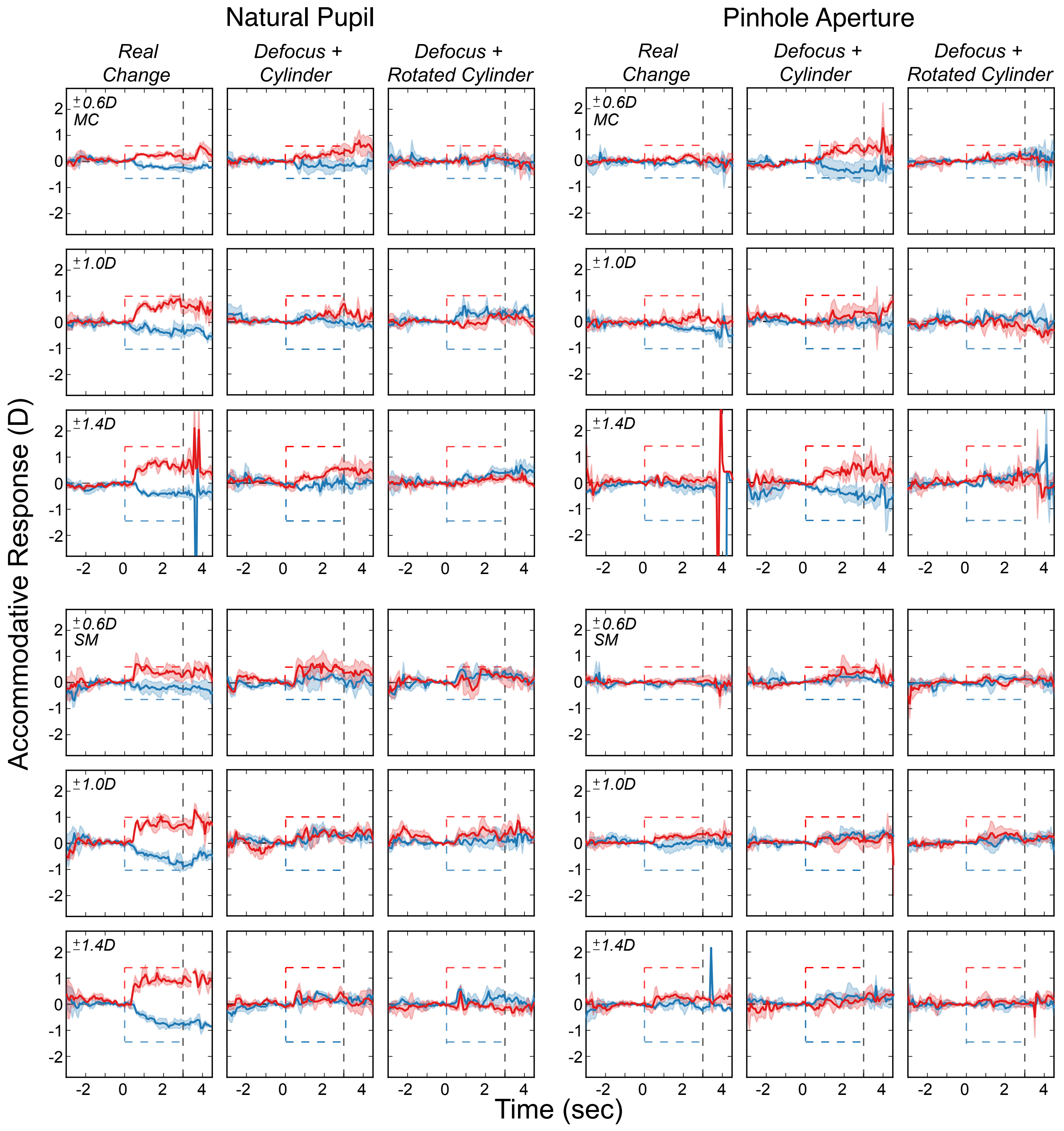

Figure S8: Data from the other two subjects in the astigmatism experiment. The format is the same as Figs. 16 and 17. Left column: responses with natural pupil. Right column: responses with a pinhole aperture. Top row is subject MC who has significant astigmatism. Bottom row is SM who has minimal astigmatism.

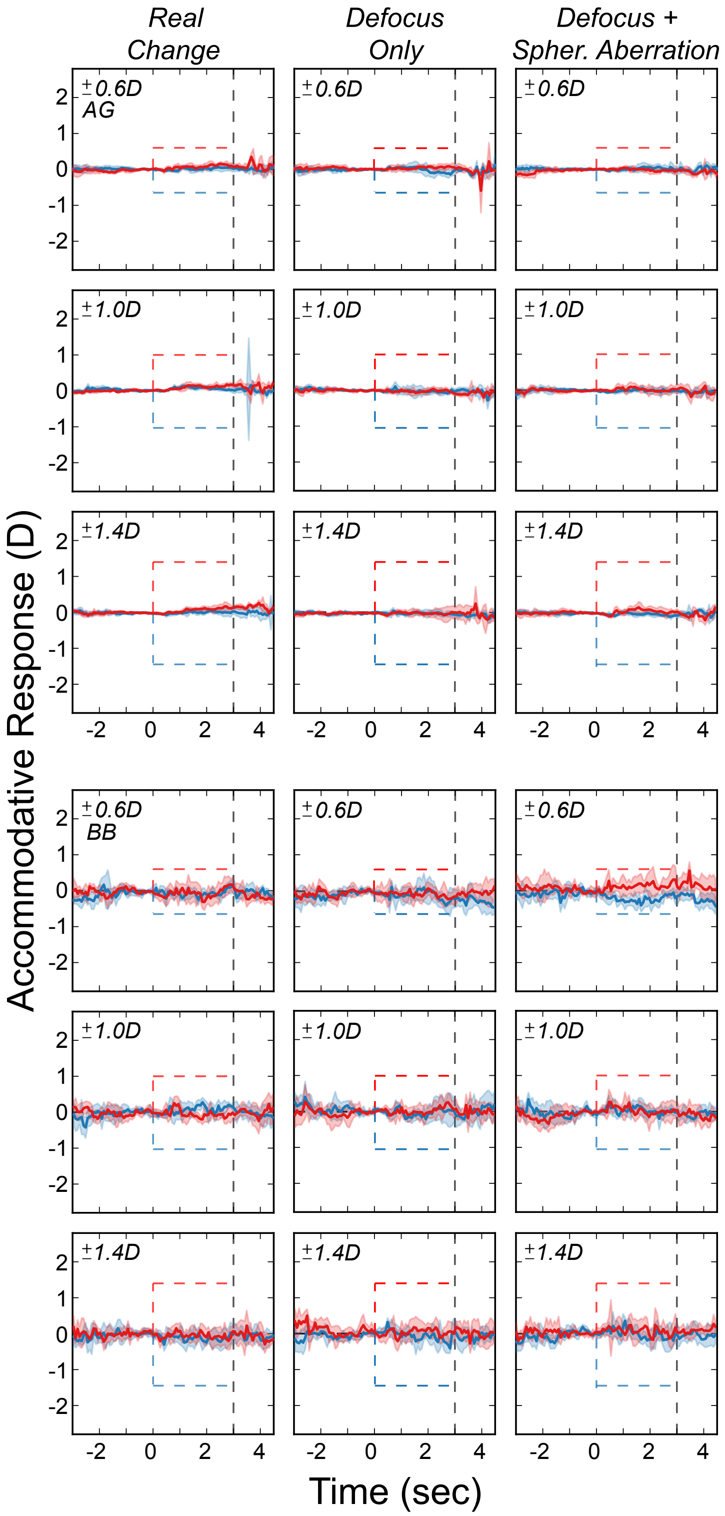

Figure S9: Data from the other two subjects in the spherical aberration experiment. The format is the same as Fig. 18. Top set of data is subject AG and bottom set is BB.

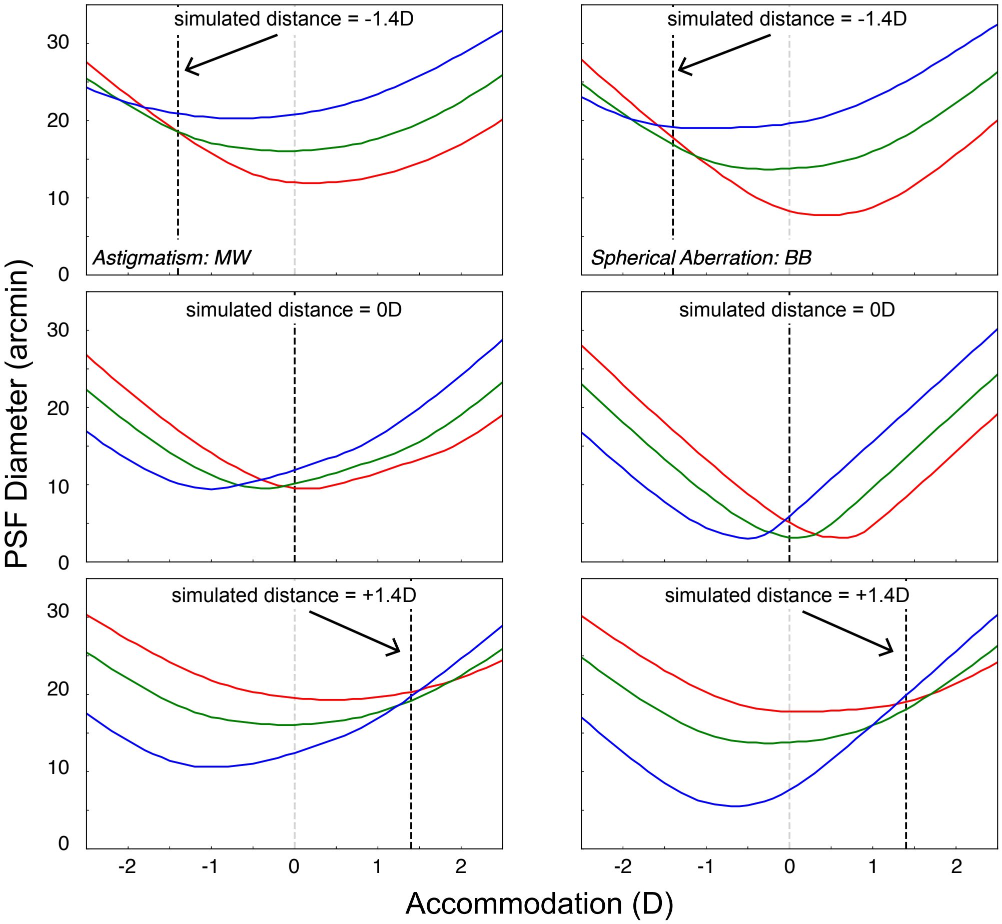

Figure S10: Retinal PSF diameters for the R, G, and B primaries generated by different combinations of simulated focal distance and accommodative response. Compared to Fig. 19, the eye model has now incorporated 21 Zernike modes for subjects MW (left) and BB (right). Top, middle, and bottom panels show those diameters for simulated distances of -1.4, 0, and $+1.4$D, respectively. Each panel plots the diameter of the PSF at the retina as a function of accommodation. The diameters were calculated using encircled energy. The red, green, and blue curves are those diameters for the R, G, and B primaries of our display. The dashed black lines represent the simulated focal distances. The dashed gray lines represent the nominal accommodative distance at stimulus onset. These curves are very similar to those in Fig. 19 and show that incorporating other aberrations has little effect.
